# Old Drugs, New Targets: Molecular Docking Insights into Repurposing Proton Pump Inhibitors for the TLR4/MD-2, NLRP3, and C5aR1 Axes in Autoimmune Liver Diseases

**DOI:** 10.3390/ph19101581

**Published:** 2026-10-06

**Authors:** Mehmet Yıldız, Erdi Şeker, Engin Altınkaya, Alireza Poustforoosh, Burak Tüzün, Hilmi Ataseven

**Affiliations:** 1 Division of Gastroenterology, Department of Internal Medicine, Faculty of Medicine, Sivas Cumhuriyet University, Sivas 58140, Türkiye; drmehmetyildiz@yahoo.com (M.Y.); altinkayaengin@gmail.com (E.A.); 2 Departmant of Gastroenterology, Sivas Numune Hospital, Sivas 58140, Türkiye; erdiseker@cumhuriyet.edu.tr; 3 Medicinal and Natural Products Chemistry Research Center, Shiraz University of Medical Sciences, Shiraz 7134853734, Iran; 4 Department of Chemistry, Faculty of Science, Sivas Cumhuriyet University, Sivas 58140, Türkiye; theburaktuzun@yahoo.com

**Keywords:** proton pump inhibitors, drug repurposing, autoimmune liver diseases, molecular docking, NLRP3 Inflammasome, TLR4/MD-2, C5aR1

## Abstract

**Background/Objectives**: Autoimmune liver diseases (AILDs), including autoimmune hepatitis, primary biliary cholangitis, and primary sclerosing cholangitis, may include treatment-resistant cases and therefore require new therapeutic strategies. This in silico study evaluated proton pump inhibitors (PPIs) across three pathophysiologically complementary inflammatory targets relevant to hepatic immune injury: TLR4/MD-2, representing upstream innate immune sensing; NLRP3, representing inflammasome-mediated inflammatory amplification; and C5aR1, representing complement-dependent leukocyte recruitment. **Methods**: The binding profiles of four PPIs (rabeprazole, lansoprazole, omeprazole, and pantoprazole) were analyzed by molecular docking against TLR4/MD-2 (3FXI), NLRP3 (7VTP and 7PZC), and C5aR1 (6C1R and 5O9H). TAK-242, MCC950, and avacopan were included as target-associated reference ligands solely to provide structural and protocol-specific context. Their docking scores were not used as benchmarks of relative binding affinity, inhibitory potency, or pharmacological efficacy. MM-GBSA binding free energy calculations for the best score in all proteins were performed. Molecular docking and molecular dynamics analyses indicated stable interactions for the best docking score. **Results**: PPIs showed variable but potentially meaningful docking affinities across the evaluated targets. Rabeprazole produced favorable docking scores within the investigated TLR4/MD-2 (−7.85 kcal/mol) and inactive NLRP3 (−9.10 kcal/mol) binding sites under the present docking protocol. The reference ligands TAK-242 and MCC950 were included only to provide structural and protocol-specific context, and their docking scores were not interpreted as measures of relative pharmacological potency. Among the PPIs, lansoprazole showed the most favorable docking score for the C5aR1 allosteric site (−6.93 kcal/mol). Interaction analyses suggested favorable hydrophobic contacts and substantial spatial occupancy within the investigated binding pockets; however, cross-target docking compatibility may partly reflect nonspecific hydrophobic accommodation rather than genuine polypharmacology, and whether these predicted interactions produce target-specific functional effects requires experimental validation. MM-GBSA analysis supported the predicted stability of the rabeprazole–NLRP3 (7PZC) complex (ΔG_bind_ = −56.97 kcal/mol). **Conclusions**: These findings prioritize rabeprazole and lansoprazole as PPI scaffolds showing favorable predicted interactions across several inflammation-related target structures within the applied computational framework. These results are hypothesis-generating and do not establish target inhibition, target engagement, or therapeutic efficacy. The results are hypothesis-generating and warrant experimental studies to determine whether these predicted interactions translate into target engagement or modulation of inflammatory signaling in AILDs.

## 1. Introduction

Autoimmune liver diseases (AILDs), including autoimmune hepatitis (AIH), primary biliary cholangitis (PBC), and primary sclerosing cholangitis (PSC), are characterized by the loss of immune tolerance and ongoing necroinflammatory liver injury. The etiopathogenesis of these complex conditions is driven by genetic predisposition, environmental triggers, and, crucially, the dysregulation of both innate and adaptive immune responses [1,2,3]. Current therapies largely rely on corticosteroids and immunosuppressive agents (e.g., azathioprine); however, their long-term use is associated with significant toxicity, and a substantial proportion of patients remain non-responsive to these treatments [4,5]. Therefore, developing safe and selectively targeted therapeutic approaches is a strategic clinical need [3,6].

At the cellular level, the recognition of damage-associated molecular patterns (DAMPs) and pathogen-associated molecular patterns (PAMPs) plays a pivotal role in the hepatic inflammatory cascade. In this context, Toll-like receptor 4 (TLR4) functions as a primary initiator of Kupffer cell and hepatic stellate cell activation in response to bacterial endotoxins (e.g., lipopolysaccharides, LPS) translocated via the gut–liver axis. The activation of the TLR4/MD-2 complex triggers a robust proinflammatory cytokine cascade, thereby exacerbating hepatic injury [7,8,9]. Furthermore, the activation of the NOD-like receptor protein 3 (NLRP3) inflammasome serves as a critical intracellular platform for the chronicity of inflammation and progression to fibrosis. NLRP3 activation promotes the maturation of potent proinflammatory cytokines, such as interleukin-1β (IL-1β) and IL-18, inducing pyroptosis and further stellate cell activation [10,11]. Similarly, the potent anaphylatoxin complement component 5a (C5a) and its receptor C5aR1 promote an increase in neutrophil and monocyte infiltration to worsen autoimmune liver injury [12]. Importantly, TLR4/MD-2, NLRP3, and C5aR1 were selected together because they represent complementary rather than redundant stages of the hepatic innate inflammatory response. TLR4/MD-2 functions predominantly as an upstream pattern-recognition system that senses gut-derived LPS and initiates inflammatory signaling in Kupffer cells and hepatic stellate cells. NLRP3 acts downstream as an intracellular amplification platform that promotes IL-1β/IL-18 maturation, pyroptosis, and fibrosis-associated signaling. In contrast, C5aR1 represents a complementary complement-dependent axis that contributes to neutrophil and monocyte recruitment and thereby sustains inflammatory-cell infiltration and tissue injury. Thus, the simultaneous evaluation of these three structurally unrelated targets was designed to interrogate three mechanistically distinct components of hepatic inflammation—initiation, intracellular amplification, and immune-cell recruitment—rather than multiple receptors belonging to a single signaling pathway.

This multi-axis design is particularly relevant to AILDs because hepatic inflammation is not driven by a single receptor or signaling cascade. Instead, microbial sensing, inflammasome activation, complement signaling, and leukocyte recruitment interact to maintain chronic inflammatory injury. Therefore, examining PPIs across these three targets within the same computational framework enables assessment of whether their previously reported pleiotropic anti-inflammatory effects might be associated with target-dependent structural compatibility at more than one inflammatory checkpoint. Importantly, such cross-target docking is used here only for candidate prioritization and does not establish simultaneous pharmacological modulation or true polypharmacology, since favorable interactions across multiple proteins may also arise from nonspecific physicochemical or hydrophobic compatibility with their binding pockets.

The discovery of novel drug candidates through de novo design is time-consuming and cost-intensive. Thus, drug repurposing—the strategy of investigating approved drugs with established safety and pharmacokinetic profiles for new therapeutic indications—has emerged as a highly rational approach [13]. Beyond their widely recognized acid-suppressive properties, proton pump inhibitors (PPIs) have recently garnered significant attention for their pleiotropic immunomodulatory and antioxidant effects [14]. Structurally, PPIs share a benzimidazole core, a “privileged scaffold” in medicinal chemistry that enables interactions with diverse biological targets [15,16].

Previous experimental and computational studies provide evidence that PPIs can exert biological effects beyond gastric H^+^/K^+^-ATPase inhibition. Experimental studies have reported that lansoprazole can modify LPS-stimulated TLR4 signaling, while omeprazole has been associated with attenuation of the TLR4/NF-κB/NLRP3 inflammatory axis in experimental models. In parallel, molecular docking has been applied to PPIs in other biological contexts, including their interactions with metabolic enzymes, plasma transport proteins, and, more recently, microbial protein targets. Collectively, these studies support the concept that PPIs can interact with proteins beyond their canonical gastric target. However, the available computational literature is fragmented across unrelated targets and biological contexts, and it does not provide a systematic structure-based assessment of clinically used PPIs across the TLR4/MD-2, NLRP3, and C5aR1 inflammatory axes relevant to hepatic immune injury. Despite these advances, an important computational gap remains at the intersection of PPI repurposing and multi-axis inflammatory target assessment. Previous studies have either investigated the non-gastric anti-inflammatory effects of PPIs experimentally or have used structure-based methods to study individual inflammatory targets with purpose-designed inhibitors or unrelated anti-inflammatory compounds. To the best of our knowledge, clinically used PPIs have not been systematically evaluated within a single computational framework across TLR4/MD-2, NLRP3, and C5aR1 in the specific context of autoimmune liver inflammation.

The present study was therefore designed to advance previous computational work in three respects. First, rather than examining a single inflammatory protein, we evaluated three structurally unrelated but pathophysiologically complementary targets representing inflammatory initiation (TLR4/MD-2), intracellular amplification and pyroptotic signaling (NLRP3), and complement-dependent leukocyte recruitment (C5aR1). Second, multiple structural states and binding environments were included, namely the MD-2 hydrophobic pocket, the active and ligand-bound inactive NLRP3 conformations (7VTP and 7PZC), and the allosteric and extra-helical C5aR1 regions (6C1R and 5O9H). Third, four clinically established PPIs—rabeprazole, lansoprazole, omeprazole, and pantoprazole—were evaluated under a common docking protocol to identify target- and pocket-dependent interaction patterns. These compounds were selected as a focused set of established PPIs that share the characteristic substituted benzimidazole–pyridine pharmacophore while exhibiting structural variation in their peripheral substituents, thereby enabling an initial within-class comparison within a manageable and consistently treated computational framework. The present study was designed as a hypothesis-generating repurposing analysis rather than as an exhaustive screening of the entire PPI or benzimidazole chemical space. Consequently, other clinically used PPIs and broader benzimidazole analogs were outside the predefined scope of the present analysis. Their exclusion should not be interpreted as evidence of lower relevance or weaker interaction potential, and systematic expansion to additional PPI members and benzimidazole derivatives represents an important direction for subsequent structure–activity studies. Molecular docking and MM-GBSA were used as hypothesis-generating tools for candidate prioritization rather than as evidence of simultaneous inhibition or therapeutic efficacy.

In this study, we hypothesized that PPIs may exhibit favorable predicted binding profiles within selected active and allosteric pockets of TLR4/MD-2, NLRP3, and C5aR1. Through molecular docking and MM-GBSA calculations, we aimed to characterize these putative ligand–protein interactions and prioritize PPI scaffolds for subsequent experimental investigation. Through an in silico molecular docking and MM-GBSA-based thermodynamic assessment, we aimed to provide a molecular rationale for further evaluating PPIs as potential anti-inflammatory and immunomodulatory candidates in AILDs and to elucidate the structural basis of their pleiotropic effects. Accordingly, the novelty of this study lies not in applying molecular docking to inflammatory proteins per se, but in integrating PPI repurposing, cross-target comparison, multiple receptor conformations, and AILD-relevant inflammatory biology within a unified structure-based framework.

## 2. Results

**Figure 1:**
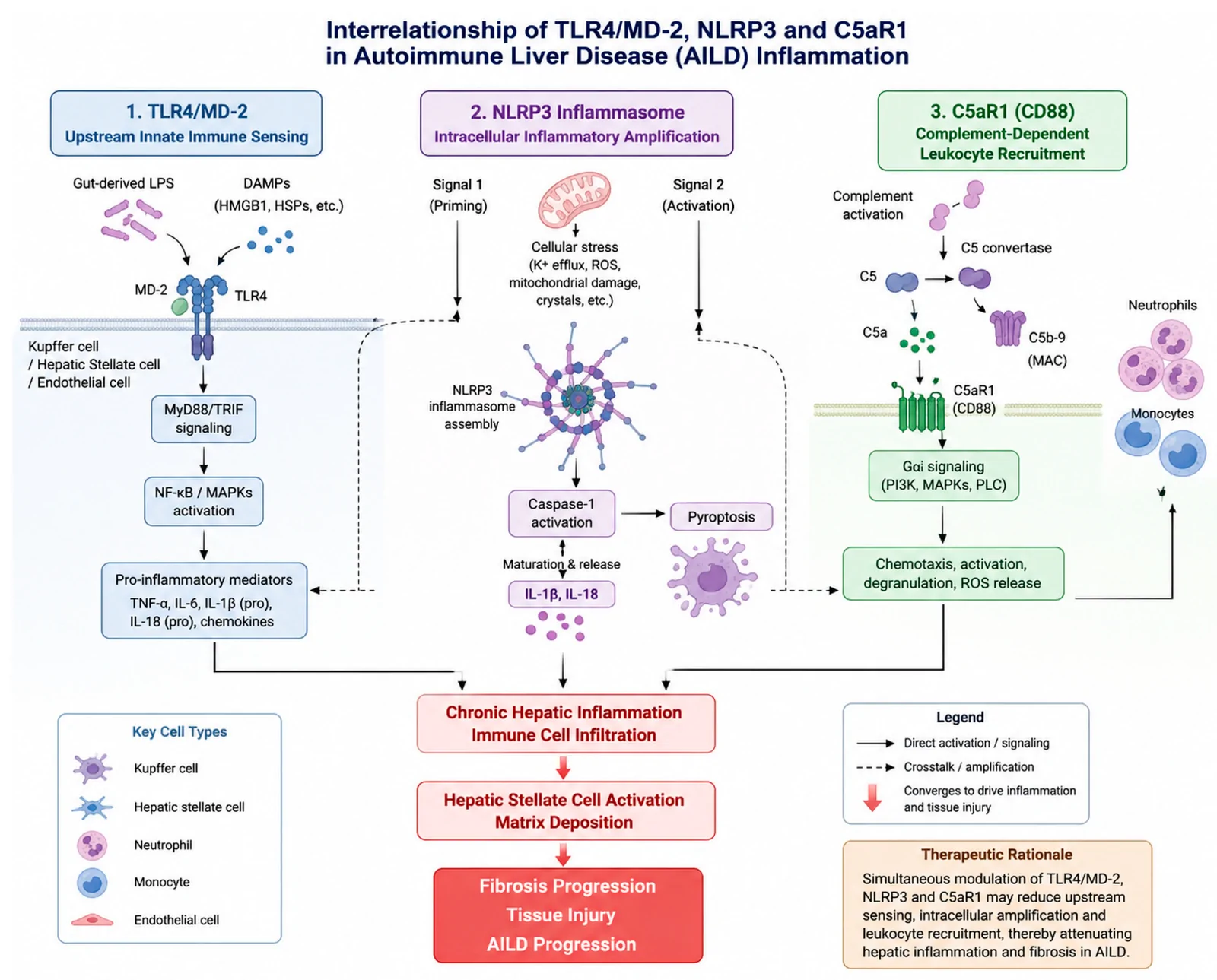
Biological diagram showing the relationship between TLR4/MD-2, NLRP3, and C5aR1 in AILD inflammation.

The docking scores, docking-derived interaction profiles, and MM-GBSA energy components of four proton pump inhibitors against TLR4/MD-2 (3FXI), NLRP3 (7VTP and 7PZC), and C5aR1 (6C1R and 5O9H) are summarized in Table 1, Table 2, Table 3, Table 4, Table 5 and Table 6. The results are presented according to each target protein, with comparative evaluation against the corresponding reference candidate ligands. Biological diagram showing the relationship between TLR4/MD-2, NLRP3, and C5aR1 in AILD inflammation in Figure 1.

### 2.1. Interactions with the TLR4/MD-2 Complex (3FXI)

Molecular docking analyses targeting the TLR4/MD-2 complex (PDB: 3FXI) showed that the tested PPIs, with the exception of pantoprazole, yielded favorable docking scores within the selected binding pocket. Detailed docking scores and docking-derived energy components are provided in Table 1. Rabeprazole demonstrated the highest predicted docking score (−7.85 kcal/mol), followed by lansoprazole (−7.41 kcal/mol) and omeprazole (−7.05 kcal/mol). Rabeprazole, lansoprazole, and omeprazole yielded docking scores of −7.85, −7.41, and −7.05 kcal/mol, respectively, within the selected MD-2 pocket under the present docking protocol. TAK-242 was included only as a target-associated pharmacological reference to provide structural and protocol-specific context. Because TAK-242 has a distinct experimentally established mechanism of action that is not equivalent to competitive occupation of the investigated MD-2 pocket, its docking score was not used as a benchmark for PPI potency. Accordingly, numerical differences between the docking scores of TAK-242 and the PPIs cannot be interpreted as evidence of superior binding affinity, TLR4 inhibition, or pharmacological efficacy of the PPIs.

**Figure 2:**
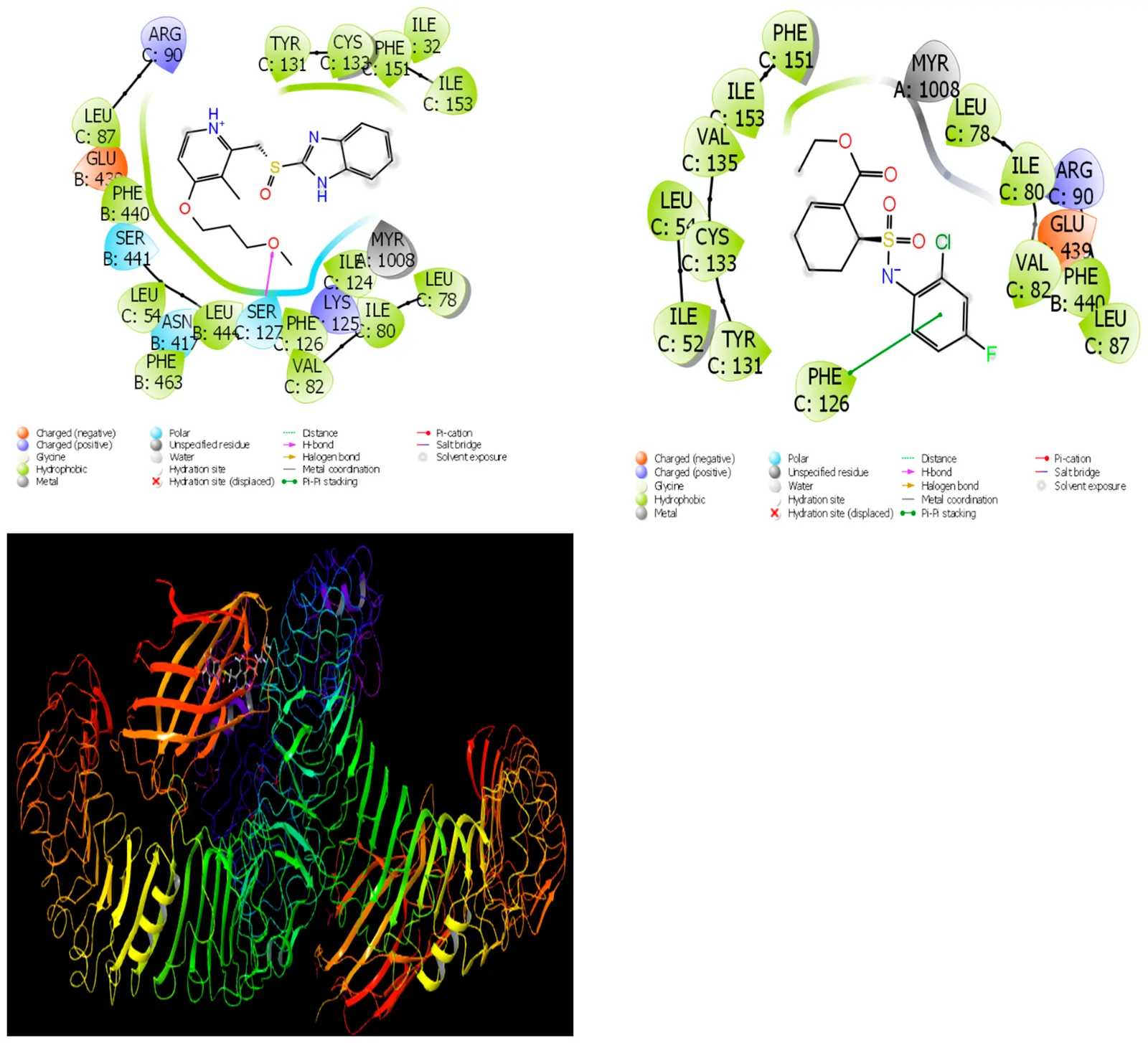
Binding orientations of rabeprazole and TAK-242 molecules at the TLR4/MD-2 (3FXI) active site. Rabeprazole is shown on the left, and TAK-242 complexes are shown on the right.

Within the computational setup used here, rabeprazole was predicted to occupy the selected MD-2 hydrophobic pocket and to contact multiple hydrophobic and polar residues. A docking pose was also generated for TAK-242 within the same computational pocket for descriptive purposes. However, because TAK-242 has a distinct experimentally established mode of action, differences between these predicted poses should not be interpreted as evidence of superior binding, inhibitory potency, or pharmacological efficacy of rabeprazole. For rabeprazole, the aromatic ring and sulfoxide backbone establish an extensive hydrophobic network involving residues PHE440, PHE463, LEU78, LEU87, ILE80, ILE124, LYS125, PHE126, and VAL82. Furthermore, TYR131 and CYS133 are optimally positioned for π-hydrophobic interactions. The protonated amine group of rabeprazole exhibits a potential electrostatic interaction with ARG90, while GLU439 and SER441 engage in polar contacts with the ligand’s heteroatoms. The flexible aliphatic chain of rabeprazole accommodates well within the pocket around LEU54, ASN417, and SER127 (Figure 2).

In contrast, the reference candidate ligand TAK-242 adopts a much more compact and restricted binding mode. Its aromatic ring forms hydrophobic contacts limited to PHE440, LEU87, VAL82, and ILE80, alongside a π–π interaction with PHE126. The predicted rabeprazole and TAK-242 poses exhibited different interaction patterns within the selected computational pocket. Rabeprazole showed predicted contacts with residues, including LEU78, ILE124, ASN417, and ARG90, whereas TAK-242 displayed a more spatially restricted interaction pattern in this particular docking setup. These differences are presented solely as structural observations and should not be interpreted as evidence of stronger binding, greater inhibitory potency, or superior pharmacological activity of rabeprazole. These numerical differences reflect only the scoring behavior of the selected docking protocol and should not be interpreted as relative binding affinity, inhibitory potency, or pharmacological efficacy (Figure 2).

### 2.2. Interactions with the NLRP3 Inflammasome (7VTP and 7PZC)

To evaluate potential interactions across different conformational states of the NLRP3 protein, two specific structures were utilized: 7VTP (active hexameric form) and 7PZC (candidate ligand-bound inactive form).

#### 2.2.1. Interaction Analyses on the Active Conformation (7VTP)

Docking against the NLRP3 (7VTP) conformation yielded scores of −6.79, −6.76, −6.34, and −5.59 kcal/mol for rabeprazole, lansoprazole, omeprazole, and pantoprazole, respectively. Rabeprazole and MCC950 exhibited distinct predicted interaction patterns within the investigated 7VTP pocket. Rabeprazole showed contacts involving PHE373, PHE520, ILE234, ILE521, LEU171, and MET523, whereas the predicted MCC950 pose involved a different set and distribution of hydrophobic and polar contacts. These pose-level differences are presented only to describe ligand–pocket accommodation within the applied docking protocol and do not establish relative binding affinity or inhibitory potency. Rabeprazole displayed the most favorable predicted interaction energy profile, underpinned by a dominant van der Waals contribution (−45.13 kcal/mol) (Table 2).

**Figure 3:**
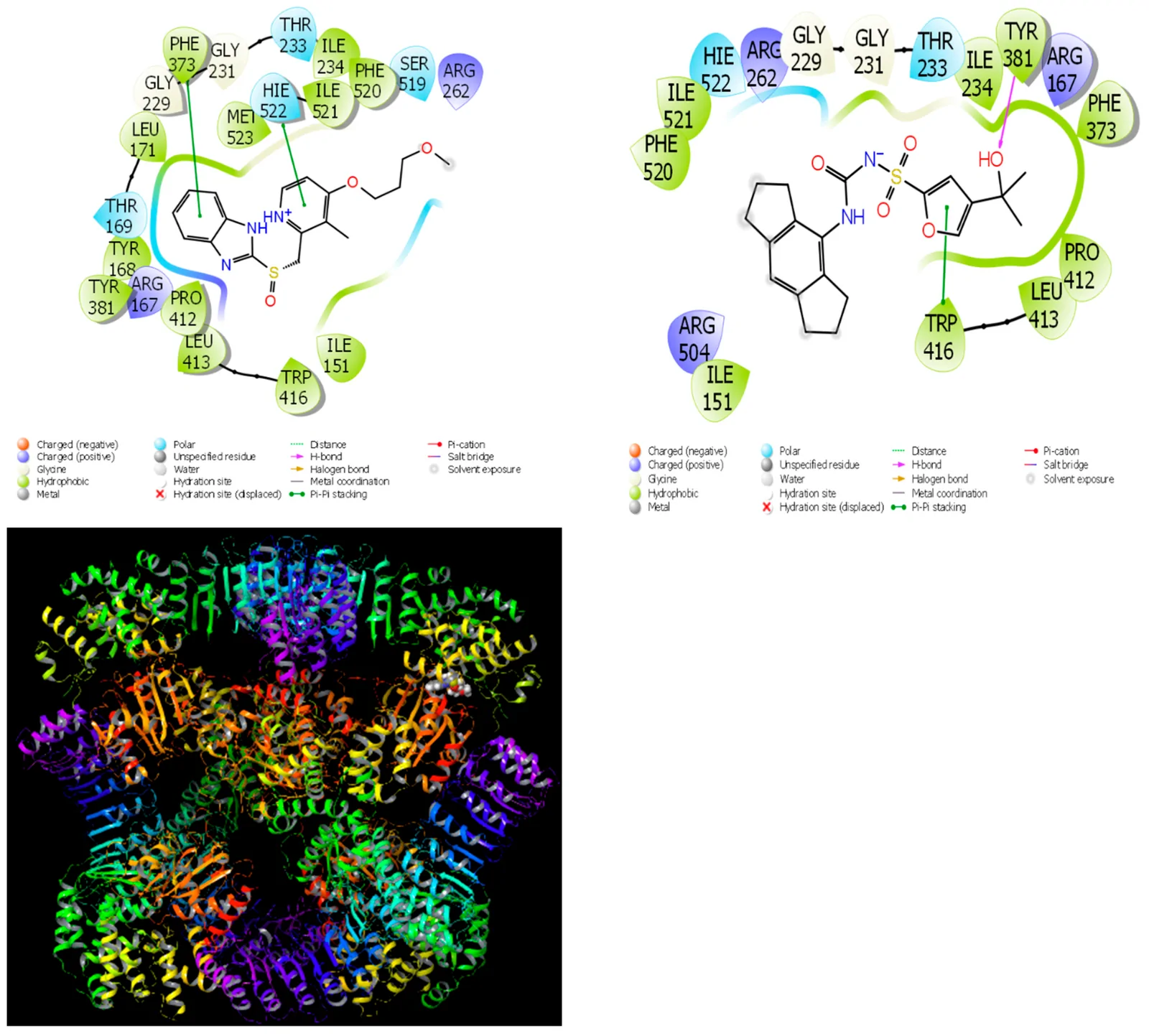
Predicted binding poses of rabeprazole and MCC950 within the investigated NLRP3 (7VTP) pocket. Rabeprazole (Docking Score: −6.79) is shown on the left and MCC950 (Docking Score: −6.25) on the right. The scores are presented only as protocol-dependent computational descriptors and should not be interpreted as measures of relative binding affinity or inhibitory potency.

Structural analysis of the 7VTP complexes indicates that both rabeprazole and MCC950 are stabilized primarily via hydrophobic interactions. The rabeprazole complex features a broad interaction interface involving PHE373, PHE520, ILE234, ILE521, LEU171, and MET523. Residues TYR168 and TYR381 facilitate π-hydrophobic interactions, while the protonated amine moiety forms hydrogen bonds and polar contacts with HIE522 and THR233. Conversely, MCC950 exhibits a more localized binding mode centered around a hydrophobic core (PHE373, ILE234, ILE381, LEU413) and relies on hydrogen bonds with THR233, GLY231, and GLY229 for polar stabilization. Within the present docking protocol, rabeprazole and lansoprazole generated the most favorable docking scores among the tested PPIs. MCC950 was retained as a structural reference only; no pharmacological ranking between MCC950 and the PPIs was inferred from the docking scores (Figure 3).

#### 2.2.2. Interaction Analyses on the Inactive Conformation (7PZC)

Docking simulations on the candidate ligand-bound inactive conformation (7PZC) yielded the highest predicted docking score values observed in this study. All tested PPIs yielded docking scores more favorable than the operational screening value of −6.0 kcal/mol used for within-study prioritization. Among them, rabeprazole (−9.10 kcal/mol) and lansoprazole (−9.04 kcal/mol) produced the most favorable docking scores within the 7PZC pocket under the present docking protocol. Importantly, the −6.0 kcal/mol value was not considered a universal activity cutoff, and compounds above or below this value should not be classified as biologically inactive or active solely on the basis of docking score. MCC950 yielded a docking score of −6.40 kcal/mol in the same computational setup. These numerical differences are reported descriptively and should not be interpreted as evidence that the PPIs possess greater binding affinity or inhibitory potency than MCC950. The favorable docking scores obtained for rabeprazole and lansoprazole within the 7PZC model were accompanied by van der Waals contributions of −40.83 and −38.72 kcal/mol, respectively. These values describe components of the protocol-dependent scoring results and should not be interpreted as evidence of greater NLRP3 inhibitory potency than MCC950 (Table 3).

**Figure 4:**
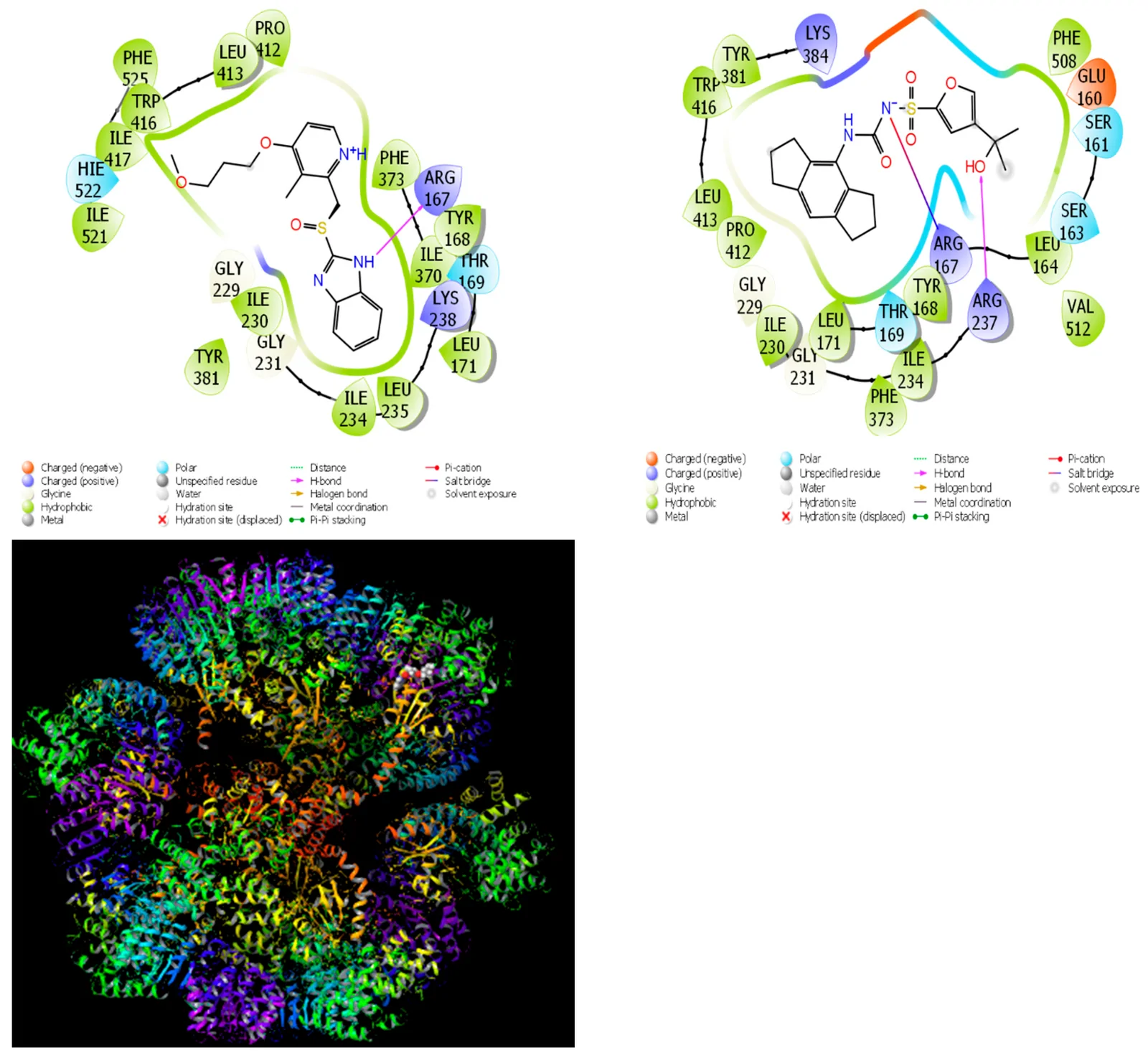
Binding orientations of rabeprazole and MCC950 molecules in the NLRP3 (7PZC) inactive pocket. Rabeprazole (Docking Score: −9.10) complexes are presented on the left, and MCC950 (Docking Score: −6.40) complexes on the right. Docking scores are shown for descriptive computational comparison only and do not represent experimentally measured binding affinities or inhibitory potencies.

The interaction diagram for rabeprazole shows a distributed predicted interaction pattern within the investigated 7PZC pocket, including hydrogen-bonding contacts with ARG167, TYR168, and LYS238, together with π–π and hydrophobic contacts involving surrounding residues. These predicted contacts characterize the docked pose only and do not indicate stronger NLRP3 inhibition than that produced experimentally by MCC950. MCC950, while forming strong charged interactions with GLU160 and LYS384, presents a much narrower hydrophobic footprint. Rabeprazole and lansoprazole produced favorable docking scores and extensive predicted contacts within the investigated 7PZC pocket. MCC950 was included as an established NLRP3 inhibitor for structural reference; however, no inference regarding relative inhibitory potency can be made from these docking-score differences (Figure 4).

### 2.3. Interactions with the C5aR1 Receptor (6C1R and 5O9H)

To assess predicted structural compatibility with the C5aR1 receptor, the allosteric pocket (6C1R) and extra-helical region (5O9H) were investigated.

#### 2.3.1. Interaction Analyses on the Allosteric Site (6C1R)

**Figure 5:**
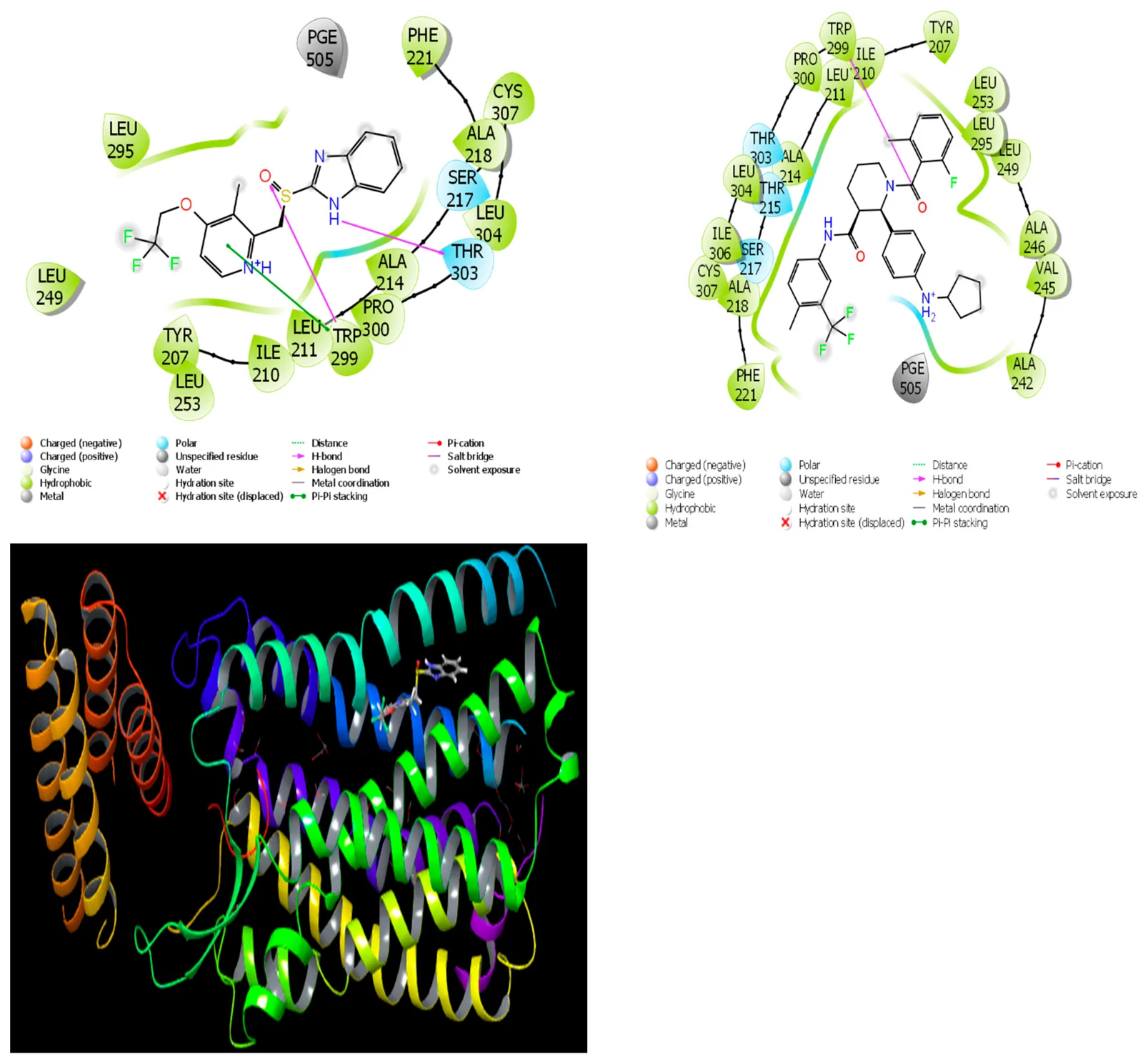
Binding orientations of lansoprazole and avacopan molecules in the C5aR1 (6C1R) allosteric pocket. Lansoprazole (Docking Score: −6.93) complexes are presented on the left, and avacopan (Docking Score: −7.94) complexes on the right.

Within the present docking protocol, avacopan produced the most favorable docking score (−7.94 kcal/mol) at the investigated allosteric site. At the extra-helical site, avacopan yielded a docking score of −8.67 kcal/mol, whereas lansoprazole produced the most favorable score among the PPIs (−6.54 kcal/mol). Among the tested PPIs, lansoprazole (−6.93 kcal/mol) and rabeprazole (−6.46 kcal/mol) produced the most favorable docking scores within the investigated 6C1R pocket under the present computational protocol (Table 4). Avacopan was included as a reference ligand for structural and protocol-specific context; therefore, differences between its docking score and those of the PPIs should not be interpreted as a ranking of binding affinity, inhibitory potency, or pharmacological efficacy. Lansoprazole’s interactions are deeply localized in the lower region of the pocket, forming key hydrogen bonds with SER217, THR303, and LEU304. Avacopan, however, spans the entire pocket, anchoring at both the top (ILE210/LEU211) and bottom (ALA242/VAL245) (Figure 5). Docking scores are presented for descriptive computational comparison only and do not represent experimentally measured binding affinities or inhibitory potencies. Notably, avacopan incurs a high internal strain energy (Glide einternal: 21.78), indicating conformational tension that is ultimately offset by massive van der Waals contacts (−46.00 kcal/mol) (Table 4).

#### 2.3.2. Interaction Analyses on the Extra-Helical Region (5O9H)

**Figure 6:**
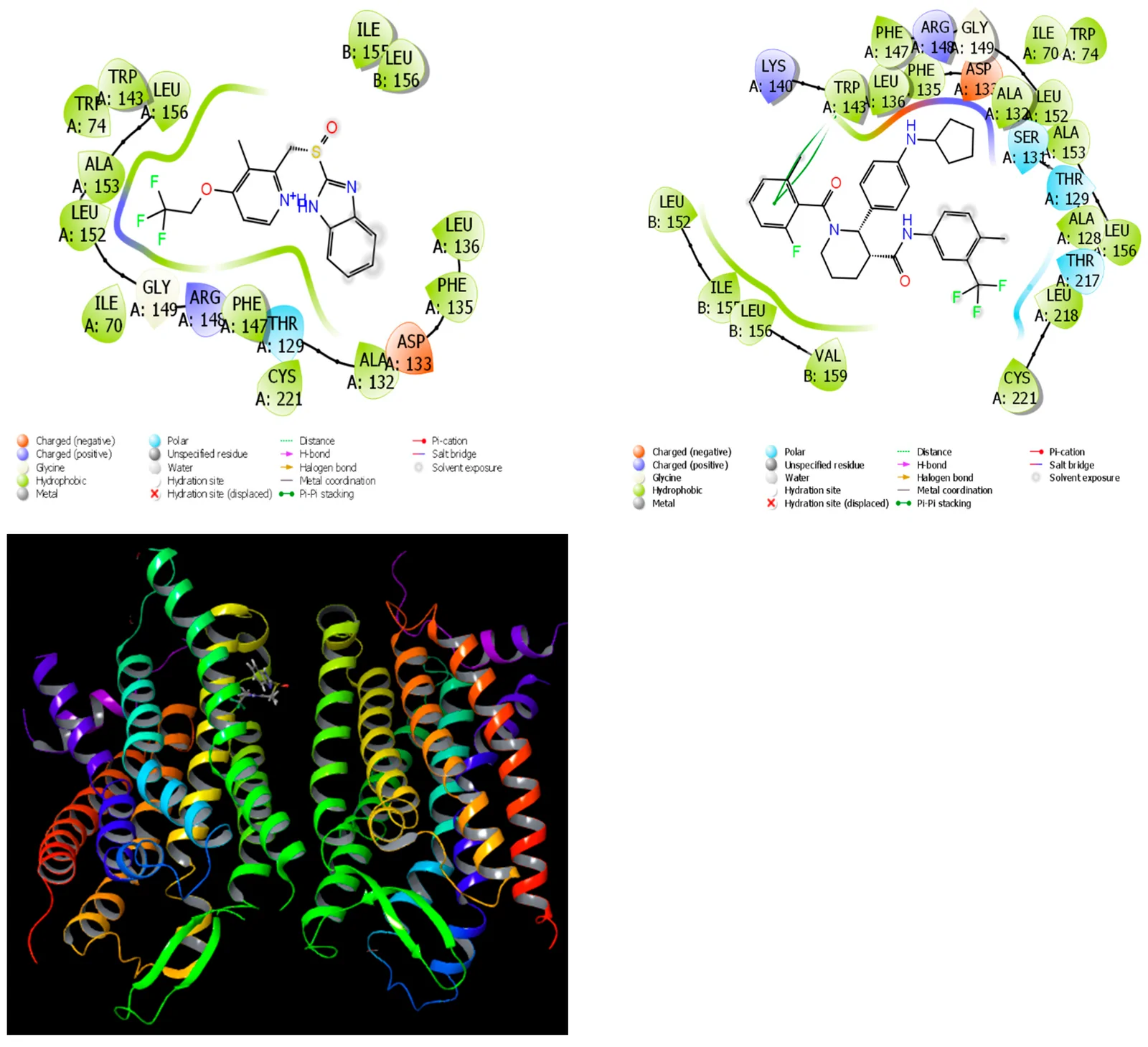
Binding patterns of lansoprazole and avacopan in the C5aR1 (5O9H) extra-helical region. Lansoprazole (Docking Score: −6.54) complex is on the left, and avacopan (Docking Score: −8.67) complex is on the right.

At the extra-helical region (5O9H), avacopan yielded a docking score of −8.67 kcal/mol, whereas lansoprazole produced the most favorable docking score among the tested PPIs (−6.54 kcal/mol) (Table 5). Structural analysis indicated that the predicted avacopan pose engaged residues across a broader region of the investigated cavity (TRP143 to CYS221), whereas the predicted lansoprazole pose showed more localized contacts involving ARG148, THR129, and ASP133 (Figure 6). These differences are reported as protocol-dependent computational observations and should not be interpreted as evidence of relative binding affinity, inhibitory potency, or pharmacological efficacy.

### 2.4. Binding Free Energy (MM-GBSA) Analysis

To determine the energetic favorability of the top-ranked ligand–protein complexes detected by molecular docking, we performed MM-GBSA calculations and summarized the binding free-energy components in Table 6. All the complexes have negative total binding free energy (ΔG_bind_), which means that they are energetically favorable for the calculations. Among the five top-ranked PPI–target complexes subjected to MM-GBSA analysis, the rabeprazole–NLRP3 (7PZC) complex yielded the most negative calculated ΔGbind value (−56.97 kcal/mol), followed by rabeprazole–TLR4/MD-2 (3FXI; −53.96 kcal/mol), lansoprazole–C5aR1 (6C1R; −43.90 kcal/mol), lansoprazole–C5aR1 (5O9H; −43.39 kcal/mol), and rabeprazole–NLRP3 (7VTP; −36.69 kcal/mol). These values indicate differences in the predicted energetic behavior of the modeled complexes within the applied MM-GBSA framework and should not be interpreted as experimentally determined binding affinities or as a ranking of biological inhibitory potency. The Lansoprazole complexes with C5aR1 have negative binding free energy of −43.90 kcal/mol for 6C1R and −43.39 kcal/mol for 5O9H, while rabeprazole binds to the active NLRP3 (7VTP) and has negative binding free energy (ΔG_bind_) of −36.69 kcal/mol. Our results suggest that the target and conformational states of the ligand–protein complexes are highly correlated in the predicted energetic behavior of ligand–protein complexes.

The MM-GBSA energies appear to be decoupled by large differences in the contribution of the total binding energies. The 7PZC-rabeprazole complex had more solvation (ΔG_bind_,SolvGB = −162.23 kcal/mol) and van der Waals (ΔG_bind,vdW = −46.64 kcal/mol) as well as lipophilic (ΔG_bind_,Lipo = −20.30 kcal/mol) contributions to the predicted binding profile, even though the Coulombic contribution was 168.65 kcal/mol. The other complexes had different balances of solvation, electrostatic, packing and internal contact. Thus, the favorable total ΔG_bind_ values seem to be a result of different combinations of the energy in the different complexes, not from one dominant interaction mechanism.

### 2.5. MD Simulation

While the receptor proteins are assumed to be stable and stiff, the ligand molecules can move and alter their attachment to the receptor in molecular docking simulations. To properly evaluate how the medicine interacts with the protein and influences its energy and mobility, the protein must be highly mobile. As MD simulation is a useful tool for evaluation of candidate ligands [17,18], 100 nanosecond molecular dynamics simulation was performed to better understand the protein’s appearance and movement. 

### 2.6. ADME/T Result

The ADME parameters in Table 7 show that omeprazole, pantoprazole, rabeprazole, avacopan, and lansoprazole have very different pharmacokinetics. Even though all these compounds are in the 130–725 Da range for QikProp in terms of molecular weight, avacopan is the largest molecule in the group of molecules with 582 Da molecular weight, with omeprazole having 345 Da molecular weight. Pantoprazole, rabeprazole and lansoprazole have very similar molecular sizes with 383, 359 and 369 Da molecular weight, respectively. This trend is not only reflected in molecular volume values; avacopan has 1675 Å^3^ and lansoprazole has 551 Å^3^. Volumes calculated for omeprazole, pantoprazole and rabeprazole are similar (1064, 1084 and 1145 Å^3^, respectively). The volumes in omeprazole, pantoprazole and rabeprazole are also very similar, and these three proton pump inhibitors have similar geometric sizes [19].

When looking at the dipole moment values, an indicator of molecular polarity, values of 3.2, 5.4, 3.3, and 2.4 D were obtained for omeprazole, pantoprazole, rabeprazole, and avacopan, respectively, all within the 1.0–12.5 D reference range, whereas the value of 26.2 D for lansoprazole is above the upper limit of the reference range, indicating that this molecule has a much more pronounced charge separation than the other molecules [20]. However, further verification of this unusual value, in terms of the specific structure/protonation state used, would be useful. The total solvent accessible surface area (SASA) is highest for avacopan at 870 Å^2^ and lowest for lansoprazole at 303 Å^2^. Omeprazole, pantoprazole and rabeprazole have a moderate to similar profile with values of 600, 621 and 650 Å^2^, respectively. Similarly, avacopan has the highest QPpolrz value at 59.9 Å^3^, followed by rabeprazole at 37.2 Å^3^, pantoprazole at 35.5 Å^3^, omeprazole at 35.0 Å^3^ and lansoprazole at 15.9 Å^3^. All these values are in the 13–70 Å^3^ range.

In terms of hydrogen bonding, avacopan has the largest number of hydrogen bond donors in the series, having two donor sites; omeprazole, pantoprazole and rabeprazole have one and lansoprazole has zero. The number of hydrogen bond acceptors was nine for rabeprazole, eight for omeprazole and pantoprazole, seven for avacopan and four for lansoprazole. Rabeprazole stands out in the series in terms of the number of functional groups that can form polar interactions. On the other hand, the globularity parameter is 0.8 for omeprazole, pantoprazole, rabeprazole and avacopan, which is within the range of 0.75–0.95, while lansoprazole is outside that range. This shows that the conformation used for lansoprazole has a very different shape from the others.

Lipophilicity-related QPlogPo/w values for omeprazole, pantoprazole, rabeprazole and lansoprazole are 2.2, 2.8, 2.5 and 1.2 within the reference range of −2.0–6.5. Avacopan is far above the upper limit of the reference range with a value of 7.2, showing that it is the most lipophilic compound in the series. This is in line with our estimate of low aqueous solubility for avacopan. In fact, we found the value of QPlogS for avacopan to be −9.3 and CIQPlogS to be −9.5, both far outside the reference range of −6.5–0.5. In contrast, omeprazole (−2.3/−4.0), pantoprazole (−2.9/−4.7), rabeprazole (−2.3/−3.9) and lansoprazole (−0.3/−3.9) are all within the accepted range. In terms of QPlogS, lansoprazole has the highest estimated solubility at −0.3. In contrast, avacopan’s high lipophilicity, despite being of high hydrophobic composition, was by far the lowest solubility compound in the series. Therefore, while avacopan’s hydrophobic character might be a benefit for membrane interactions, it could also be a serious drawback in formulation and solubility [21].

A reverse picture of membrane permeability is observed. For omeprazole, pantoprazole and rabeprazole, the QPPCaco values are very close to each other at 69, 68 and 69 nm/s, respectively, while avacopan has a much higher estimated CaCO-2 permeability of 2447 nm/s and lansoprazole at 5870 nm/s. A similar trend is observed for QPPMDCK for omeprazole and rabeprazole, which were at approximately 1000 nm/s, while pantoprazole was at 3301, avacopan at 6330, and lansoprazole at 6250 nm/s. This suggests that avacopan and lansoprazole have higher membrane permeability in the model than the other three compounds. However, we should also consider that the high permeability of avacopan and very low aqueous solubility should be taken together; good membrane permeability alone does not necessarily indicate a good oral pharmacokinetic profile [22].

QPlogBB values, which represent the distribution of the blood–brain barrier, were in the range −3.0–1.2 for all compounds. Lansoprazole had the highest estimated BBB distribution at 0.5, followed by avacopan at −0.1, pantoprazole at −0.3, omeprazole at −0.5 and rabeprazole at −0.7. With regard to this distribution, rabeprazole seems to have the most limited central distribution, while lansoprazole has a higher penetration potential for the BBB. The QPlogKp values of skin permeability were in the range −1.2 to −2.0; rabeprazole showed the highest estimated skin permeability at −1.2, and lansoprazole the lowest at −2.0.

In terms of ionization energy, omeprazole (8.8 eV), pantoprazole (9.0 eV), rabeprazole (8.9 eV) and avacopan (8.2 eV) are within the reference range, while the value of 6.0 eV for lansoprazole is below the 7.9–10.5 eV reference range. This indicates that lansoprazole differs from other compounds in terms of its electronic properties. Electron affinity values are between 0.0 and 0.9 eV and remain within the reference range for all molecules. We have estimated seven metabolic reactions for omeprazole, six for pantoprazole, rabeprazole and avacopan, and only one for lansoprazole. This indicates that lansoprazole has fewer metabolic conversion points in the model, while omeprazole has more metabolic conversion points [23].

QPlogKhsa values that reflect serum albumin binding were within the reference range of −0.3 for omeprazole and pantoprazole, −0.4 for rabeprazole and −0.7 for lansoprazole, whereas the value of 1.6 for avacopan is slightly above the −1.5–1.5 range. Therefore, avacopan may bind to serum albumin more strongly than other compounds. QPlogHERG values are also very different between the compounds: lansoprazole is clearly at −0.4, while omeprazole showed −5.0, pantoprazole −5.5, rabeprazole −5.9, and avacopan −6.2. The lower values for this parameter, especially for avacopan and rabeprazole, should be considered in the context of in silico toxicity estimates and cannot be interpreted as evidence that experimental cardiac safety is indeed true. Although oral absorption is generally positive, there are notable differences [24]. The percentage of human oral absorption was calculated to be 73% for omeprazole, 76% for pantoprazole, and 75% for rabeprazole, while 100% was estimated for avacopan and lansoprazole. However, the categorical “Human Oral Absorption” value was 1 for avacopan and 3 for lansoprazole, which is a bit of a difference from the 100% value for avacopan; therefore, we would like to re-check these two data sets or data transfers in the table. The values of PSA were in the reference range of 7–200 Å^2^ for all compounds. Pantoprazole was the highest value at 79 Å^2^, omeprazole and rabeprazole at 73 Å^2^, avacopan at 65 Å^2^, and lansoprazole at only 21 Å^2^, which is the lowest value for lansoprazole. The low value of PSA for lansoprazole, together with its high CaCO-2 and MDCK permeability, is in agreement with the high membrane permeability in the framework of the model.

Omeprazole, pantoprazole, rabeprazole, and lansoprazole are drug-like in terms of drug-likeness, with no violations of Lipinski’s Rule of Five [25,26]; avacopan has two violations. As for the Rule of Three [27], omeprazole and avacopan each have one violation, and the other three compounds have no violations. So pantoprazole and rabeprazole are very stable in the classical small-molecule drug-likeness, whereas avacopan has a more unstable drug-likeness profile because of its high molecular weight, high QPlogPo/w, very low predictive solubility, and two Lipinski violations. Lansoprazole presents a very good pharmacokinetic profile with high predictive membrane permeability, 100% oral absorption, low PSA, and low metabolic conversion rate, but it is very unusual compared with the other compounds because of the high dipole moment, globularity from the reference range, and low ionization energy.

## 3. Discussion

Previous studies have established that PPIs can exert biological effects beyond acid suppression, and computational approaches have also been used to investigate their interactions with several non-canonical protein targets. The present study extends this literature by applying a unified structure-based framework to four clinically used PPIs across TLR4/MD-2, two NLRP3 conformational states, and two C5aR1 binding environments relevant to hepatic inflammation. Thus, the principal novelty of the present work is the integrated cross-target and cross-conformation assessment of PPIs in the context of AILD-related inflammatory signaling, rather than the use of molecular docking for PPIs per se [7,8,9,10,11,12]. Although TLR4/MD-2, NLRP3, and C5aR1 are not liver-specific targets and are broadly involved in systemic innate immune responses, they have well-established roles in hepatic inflammatory injury through Kupffer cell activation, inflammasome signaling, neutrophil recruitment, and stellate cell activation [1,9,10,11,12,28]. Within the framework of a drug-repurposing strategy, our in silico findings indicate that these benzimidazole-derived compounds can display favorable predicted structural compatibility with several of the investigated binding environments [15,16]. However, cross-target docking alone cannot establish genuine polypharmacology or multi-target immunomodulatory activity, because favorable scores across structurally distinct proteins may partly reflect general physicochemical compatibility with hydrophobic binding cavities rather than specific target recognition. This distinction is particularly relevant for PPIs because several of the investigated binding environments contain substantial hydrophobic regions, and the docking profiles observed in this study frequently included prominent van der Waals and hydrophobic contributions. Consequently, favorable docking across multiple targets may arise, at least in part, from nonspecific hydrophobic compatibility or ligand promiscuity rather than biologically meaningful engagement of each target. The present results should therefore be interpreted as evidence of predicted structural compatibility and candidate prioritization, not as demonstration of genuine polypharmacology. Establishing target specificity will require experimental target-engagement studies together with computational and/or experimental counter-screening against unrelated proteins and alternative hydrophobic binding pockets.

Rabeprazole and lansoprazole produced favorable docking scores across several of the investigated binding sites. Reference compounds, including TAK-242 [29,30,31], MCC950 [32,33,34,35], and avacopan [36,37,38], were included to provide structural and protocol-specific context rather than to establish pharmacological potency rankings. Because these compounds differ substantially in their experimentally established mechanisms, target sites, and optimization histories, numerical docking-score comparisons should not be interpreted as evidence that PPIs are more potent than the corresponding reference inhibitors.

### 3.1. Predicted Interactions with TLR4/MD-2 and the Gut-Liver Axis

The pathological activation of the TLR4/MD-2 signaling pathway, predominantly triggered by pathogen-associated molecular patterns (PAMPs) and damage-associated molecular patterns (DAMPs), is an important contributor to hepatocellular injury in AILDs [8,9,30]. Experimental evidence indicates that PPIs can suppress LPS-induced cytokine release [14,39].

Our docking data indicate that PPIs can adopt computationally favorable poses within the investigated hydrophobic pocket of the TLR4/MD-2 complex [40,41]. Rabeprazole and lansoprazole produced docking scores of −7.85 and −7.41 kcal/mol, respectively, within the selected MD-2 cavity. TAK-242 yielded a score of −6.04 kcal/mol when computationally placed in the same pocket; however, this value is not directly comparable in pharmacological terms because TAK-242 exerts its experimentally established TLR4 inhibitory activity through a distinct mechanism. The predicted pose of rabeprazole showed extensive spatial occupancy within the MD-2 pocket, which provides a structural hypothesis for possible ligand–pocket compatibility. However, docking alone cannot determine whether such an interaction occurs under biological conditions, interferes with LPS binding, suppresses TLR4 signaling, or produces an anti-inflammatory effect. These possibilities therefore require direct experimental validation [39,42,43].

### 3.2. Predicted Interactions with the NLRP3 Inflammasome and Their Relevance to Liver Fibrosis

NLRP3 inflammasome activation is a critical mechanism driving the progression from chronic inflammation to fibrosis via pyroptosis and hepatic stellate cell activation [10,11,44]. Docking simulations on two distinct NLRP3 conformations, namely the active 7VTP and inactive 7PZC structures, showed markedly favorable docking scores for PPIs, particularly rabeprazole and lansoprazole. Rabeprazole and lansoprazole produced docking scores of −9.10 and −9.04 kcal/mol, respectively, within the inactive 7PZC conformation. MCC950, an experimentally established high-potency NLRP3 inhibitor, yielded a score of −6.40 kcal/mol under the present docking protocol. This difference is interpreted strictly as a protocol-dependent computational observation and does not indicate that the PPIs possess greater biological affinity or inhibitory potency than MCC950. We hypothesize that PPIs may act as putative molecular plugs that stabilize NLRP3 in its inactive conformation; however, this mechanism requires confirmation through dynamic simulations and experimental validation [45]. This interpretation is consistent with the hepatoprotective effects of lansoprazole reported in acute liver injury models by Nakatake et al. [43].

### 3.3. C5aR1 and Neutrophil Infiltration

Excessive production of C5a by mast cells exacerbates collateral liver damage through neutrophil recruitment via the C5aR1 receptor [44]. For allosteric (6C1R) and extra-helical (5O9H) sites on C5aR1 lansoprazole had the most favorable docking scores (−6.93 and −6.54 kcal/mol, respectively) among PPIs tested; avacopan was more negative (−7.94 kcal/mol for 6C1R and −8.67 kcal/mol for 5O9H) in the same computational protocol, but these numerical differences are protocol dependent computational results and should not be interpreted as evidence of better binding affinity, inhibitory potency or pharmacological efficacy. At the allosteric site, 6C1R avacopan had higher Glide internal strain energy (21.78 kcal/mol) than lansoprazole (1.98 kcal/mol), indicating conformational requirements of the predicted docking poses were different. Thus lansoprazole can be considered a PPI scaffold that needs to be further tested experimentally for its interaction with C5aR1 and no pharmacological ranking between lansoprazole and avacopan can be drawn from the docking results.

### 3.4. Binding Free Energy and Thermodynamic Stability

The MM-GBSA analysis provided a further energetic perspective of the docking results and favored the PPI-target complexes. Among the complexes evaluated by MM-GBSA, the rabeprazole–NLRP3 (7PZC) complex yielded the most favorable calculated ΔGbind value (−56.97 kcal/mol) within the applied computational protocol, while the rabeprazole–TLR4/MD-2 (3FXI) complex yielded a calculated ΔGbind value of −53.96 kcal/mol. These differences describe the relative energetic behavior of the modeled complexes under the present computational conditions and should not be interpreted as evidence of preferential biological target engagement, inhibitory potency, or therapeutic efficacy. Rabeprazole is significantly less favorable to the active NLRP3 conformation (7VTP) than the 3FXI-rabeprazole binding (−53.96 kcal/mol).

For C5aR1, lansoprazole has similar favorable binding free energies for the allosteric 6C1R (−43.90 kcal/mol) and extra-helical 5O9H (−43.39 kcal/mol) environments, indicating that its predicted interaction is not restricted to a single C5aR1 structure. Crucially, the MM-GBSA energy decomposition showed that the molecular sources of stabilization differed very much between the complexes. The 7PZC-rabeprazole system exhibits a strongly favorable solvation, van der Waals, and lipophilic contribution to compensate for the Coulombic component, whereas the C5aR1 complexes exhibit different electrostatic, solvation, packing, and internal contact contributions.

Overall, the MM-GBSA results complement the docking analysis and further support the computational prioritization of the rabeprazole-NLRP3 (7PZC) complex for further molecular dynamics and experimental studies. However, the values of ΔG_bind should be considered as computational estimates, not thermodynamic binding free energies or a direct marker of inhibitory potency. Thus, the results are in line with predictions for energetic favorability and candidate prioritization but do not support target inhibition or treatment. This cautious interpretation is also the focus of this paper, which defines docking and MM-GBSA as the approach for hypothesis-generating candidate prioritization.

### 3.5. Molecular Dynamic Simulation

**Figure 7:**
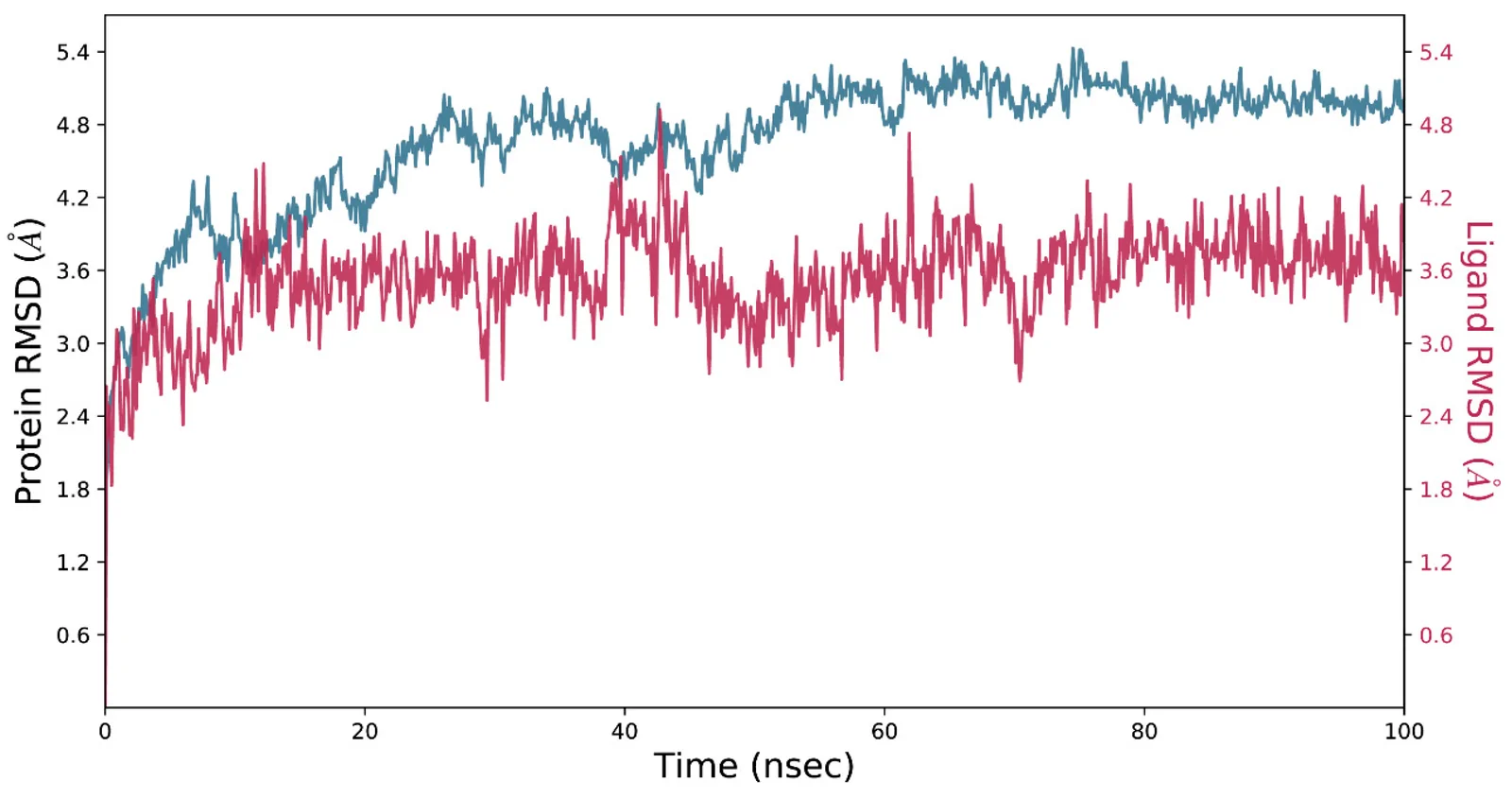
The RMSD values of ligand and protein during 100 ns. 7PZC and Rabeprazole are equilibrated at about 4.8 Å and 3.6 Å, respectively.

**Figure 8:**
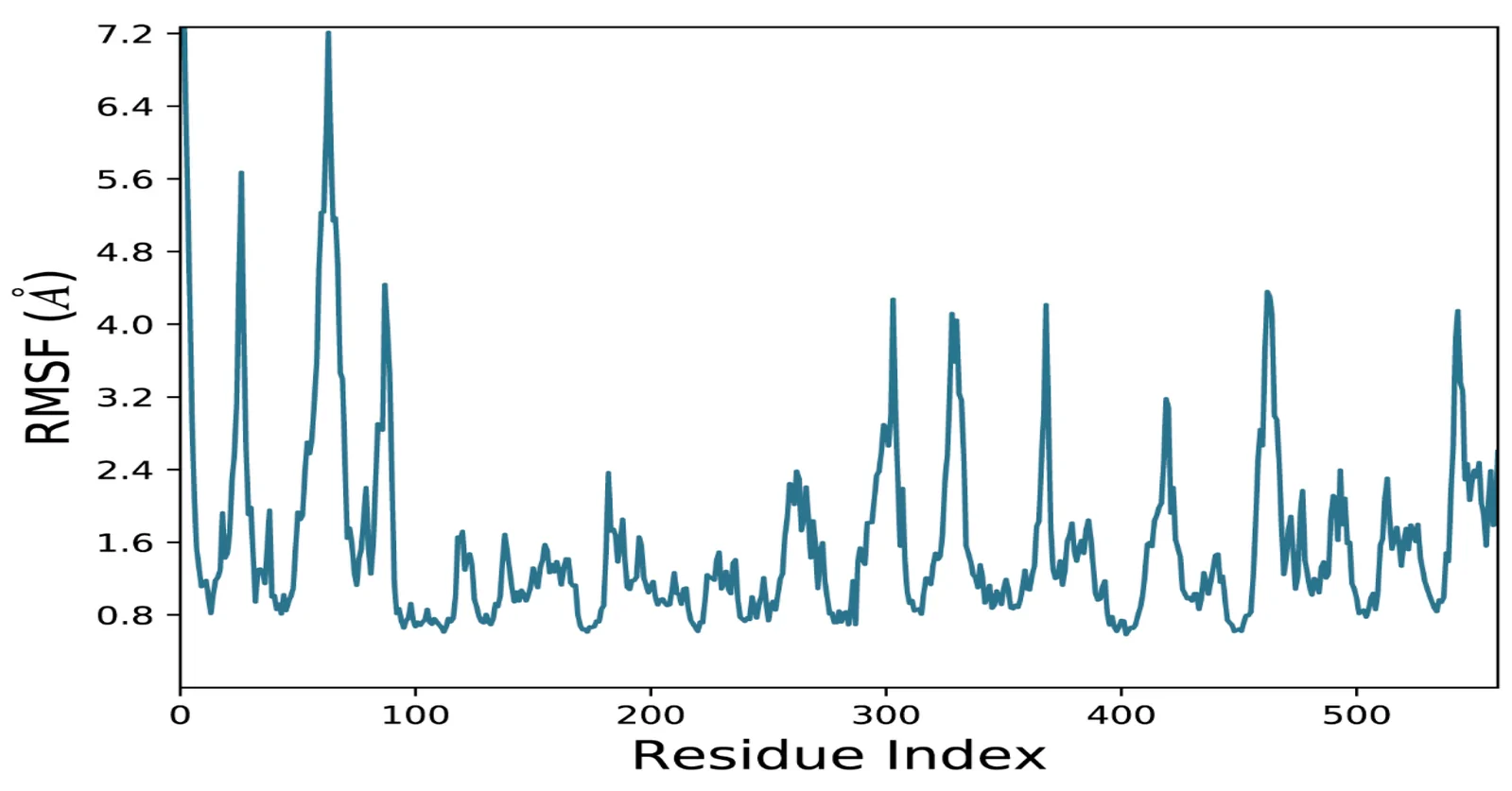
The RMSF values of the protein obtained from the 100 ns simulation.

**Figure 9:**
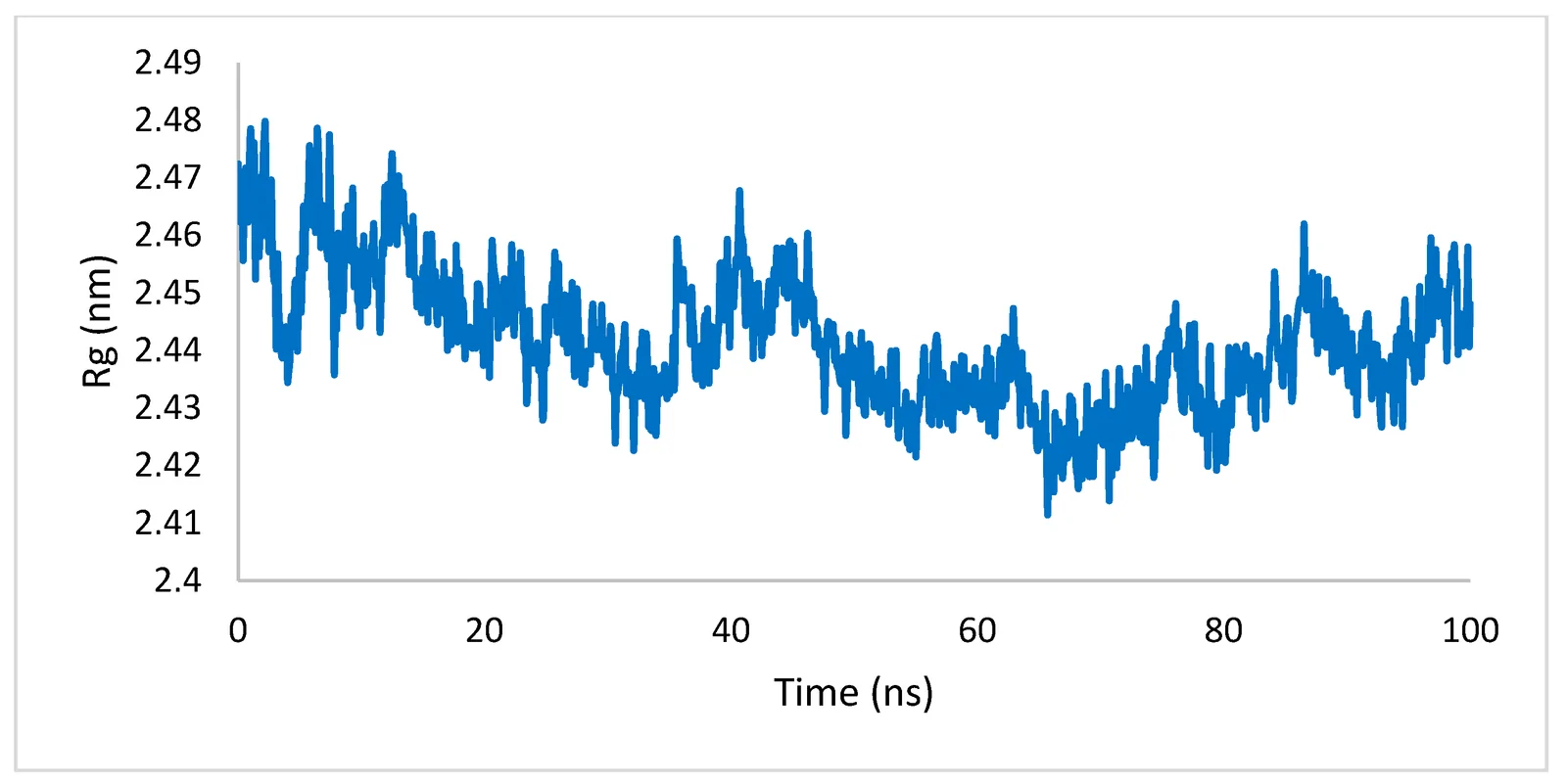
The values of the radius of gyration for the protein (7PZC) during 100 ns simulation.

**Figure 10:**
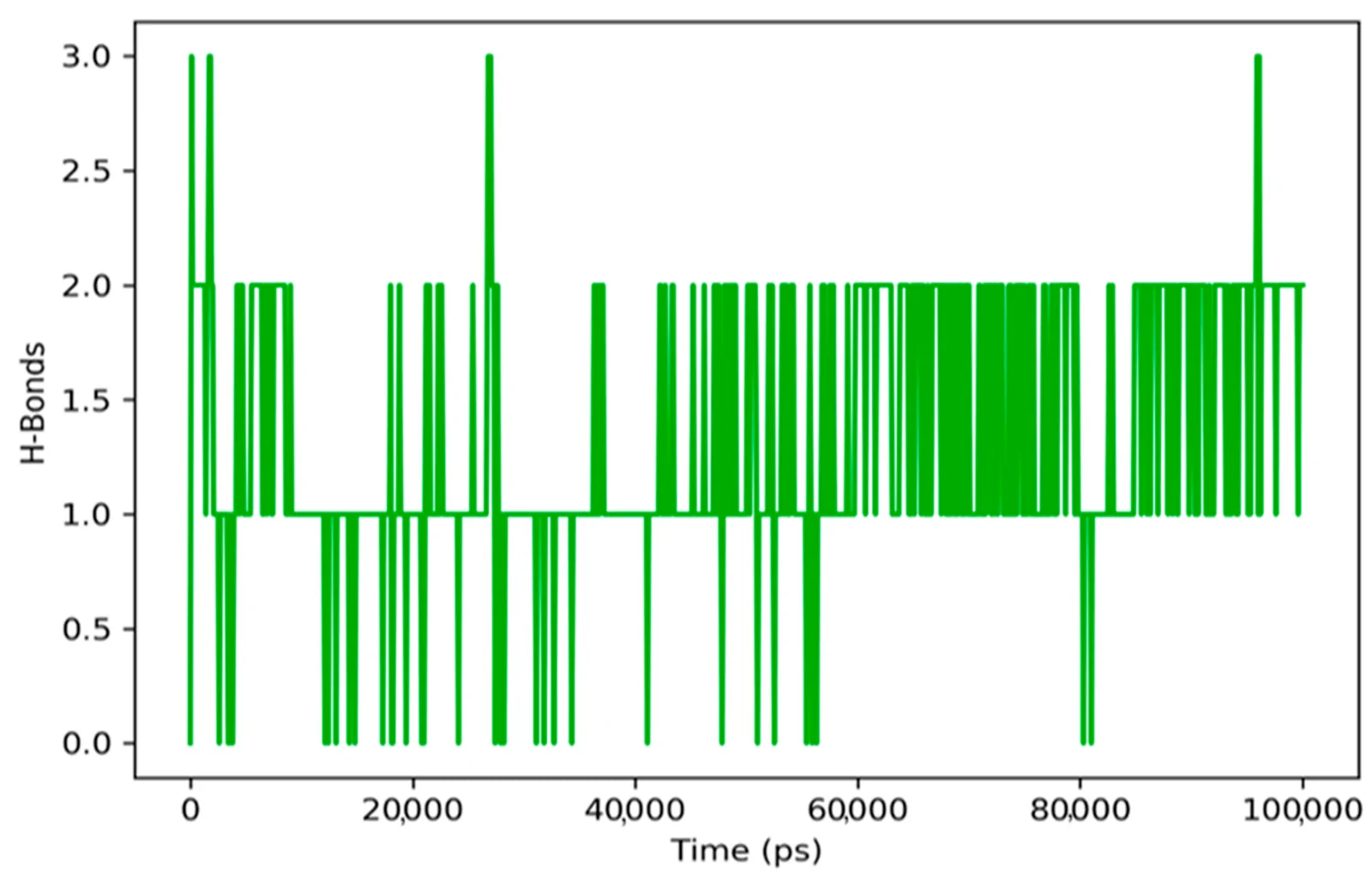
The hydrogen bonds formed between rabeprazole and 7PZC during the MD simulation.

**Figure 11:**
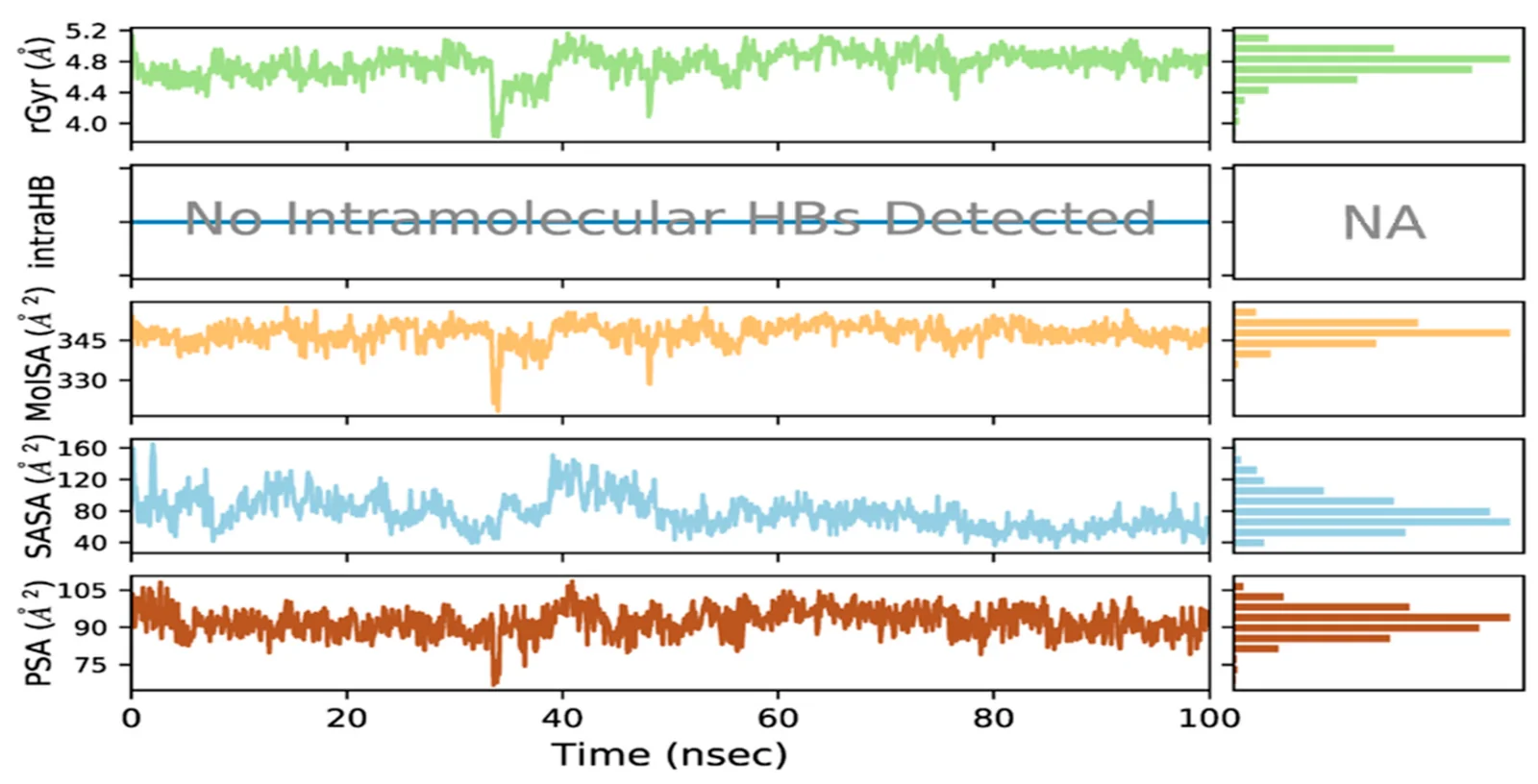
The ligand properties of rabeprazole during the simulation. The values have remained within a certain range.

We were able to comprehend the mechanics behind the protein complex’s dynamic mobility, long-term stability, and shape alterations thanks to this simulation. The radius of gyration (Rg), hydrogen bonding, root-mean-square fluctuation (RMSF), and root-mean-square deviation (RMSD) measurements were all examined in this context. Additionally, we examined the ligand’s various characteristics, including its solvent-accessible surface area (SASA). Figure 7, Figure 8, Figure 9, Figure 10 and Figure 11 illustrate this. The stability of the complex was assessed by calculating its RMSD. The changes in the structure during the course of the simulation are displayed by the RMSD variations. The average change in Cα backbone locations and ligand RMSD are calculated from the start to the finish of the simulation. The rabeprazole–7PZC complex was selected for molecular dynamics simulation because it showed the most favorable docking score among the investigated PPI–target combinations within the applied docking protocol. During the 100 ns simulation, the rabeprazole–7PZC model exhibited relatively consistent conformational behavior after the initial adjustment period, with the protein and ligand RMSD values fluctuating around approximately 4.8 and 3.6 Å, respectively. RMSF, Rg, hydrogen-bonding, and SASA analyses provided complementary information on residue flexibility, global compactness, intermolecular contacts, and ligand solvent exposure during the trajectory. These results support the persistence of the modeled rabeprazole–7PZC interaction during the simulated timescale but should not be interpreted as experimental evidence of NLRP3 binding or inhibition. The ligand and protein’s RMSD values have stabilized at around 3.6 Å and 4.8 Å, respectively. Figure 8 shows the protein’s RMSF values for this simulation. The majority of the residues have fluctuated within a small range between 0.8 Å and 2 Å, according to the RMSF data. Figure 9 displays the radius of gyration (Rg) values for the protein. These values have varied within a small range of 2.42 to 2.47 nm, as is evident. A tiny molecule can attach itself to a protein at a specific location by forming special connections, which helps the protein recognize the molecule. However, no other protein has the same ability to interact or exchange information with the chemical. Figure 10 displays the hydrogen bonds that formed between 7PZC and rabeprazole during the simulation. It is evident that the chemical and protein have formed a large number of H-bonds. The van der Waals surface representation showed that the compound’s Molecular Surface Area (MolSA) value varied constantly within a certain range. The relationship between a protein’s behavior and a ligand bound to a solvent is frequently reflected in the size of the solvent-accessible surface area (SASA). A small value improves the system’s stability. The polar surface area (PSA) of the molecule is easily accessible to the solvent. It only examines the nitrogen and oxygen atoms in the molecule. These events are the result of communication between the chemical and the protein. Figure 11 displays the aforementioned parameters. The primary objective of the molecular dynamics simulations was to evaluate the stability of the protein–ligand complex and the persistence of intermolecular interactions during the 100 ns simulation. Therefore, the dynamic behavior of the system was assessed using RMSD, RMSF, radius of gyration (Rg), SASA-related parameters, and hydrogen-bond analyses. Principal component analysis (PCA) was not performed in the present study, as the aforementioned metrics consistently indicated stable system behavior and were considered sufficient to support the conclusions drawn from the molecular dynamics investigation.

### 3.6. Pharmacokinetic and Translational Feasibility

An important consideration in interpreting the present docking results is whether the predicted interactions are pharmacokinetically achievable under clinically relevant PPI exposure [46,47]. Molecular docking scores and MM-GBSA energies should not be interpreted as experimentally determined Kd, Ki, IC_50_, or EC_50_ values, and therefore the present computational results cannot establish micromolar or nanomolar binding affinity. This distinction is particularly important when considering translational feasibility, because favorable predicted binding alone does not demonstrate that sufficient unbound drug concentrations can be achieved at the relevant target site in vivo.

PPIs are clinically established drugs with well-characterized pharmacokinetic profiles; nevertheless, their systemic exposure is influenced by absorption, metabolism, elimination, plasma protein binding, dosing regimen, and tissue distribution. Consequently, even a computationally favorable ligand–target interaction may have limited biological relevance if the free drug concentration at the target tissue remains substantially below the concentration required for target engagement. Conversely, local tissue accumulation, repeated dosing, intracellular distribution, or active metabolites may alter the relationship between total plasma concentration and target-site exposure. Therefore, plasma concentration alone cannot establish target engagement, but it represents an essential constraint when evaluating repurposing hypotheses [48].

In the present study, the favorable docking profiles of rabeprazole and lansoprazole should therefore be interpreted primarily as evidence for structural compatibility with the investigated binding pockets rather than as evidence of pharmacologically achievable inhibition. The docking score of −9.10 kcal/mol observed for rabeprazole against the NLRP3 7PZC structure, for example, cannot be directly converted into an expected micromolar or nanomolar binding constant. Similarly, the favorable MM-GBSA ΔG_bind_ value of −56.97 kcal/mol supports computational prioritization of this complex but does not establish the concentration required for biological target engagement. Establishing translational relevance will require experimental determination of binding affinity and functional potency, followed by comparison of these values with clinically achievable unbound plasma and hepatic tissue concentrations. Pharmacokinetic and ADMET considerations should therefore be integrated with docking, molecular dynamics, and experimental validation before any therapeutic inference is made [49,50].

A critical consideration for the translational interpretation of the present computational findings is whether clinically administered PPIs can achieve sufficient systemic and hepatic exposure to engage the investigated inflammatory targets. PPIs are generally characterized by relatively short plasma half-lives, extensive metabolism, and high plasma-protein binding. For example, pantoprazole reaches a reported peak plasma concentration of approximately 2.5 μg/mL following a 40 mg oral dose and is approximately 98% protein-bound, whereas lansoprazole reaches peak plasma concentrations in the low-μg/mL range following clinically relevant dosing. Rabeprazole and omeprazole are also highly protein-bound (approximately 96% and 95%, respectively) and undergo extensive metabolism. Consequently, the unbound systemic concentrations available for interaction with intracellular or membrane-associated inflammatory targets are expected to be substantially lower than total plasma concentrations. These pharmacokinetic characteristics raise an important translational uncertainty regarding whether the predicted TLR4/MD-2, NLRP3, and C5aR1 interactions can occur at clinically achievable exposures.

Importantly, the docking scores and MM-GBSA ΔGbind values calculated in the present study cannot be directly converted into Kd, Ki, IC50, or EC50 values and therefore cannot be quantitatively compared with clinical plasma or hepatic concentrations. Accordingly, the present calculations do not establish that clinically achievable PPI concentrations are sufficient to engage TLR4/MD-2, NLRP3, or C5aR1 in vivo. Establishing translational feasibility will require experimentally determined target-binding affinities and functional concentration–response data, followed by comparison with clinically achievable unbound plasma concentrations and, ideally, unbound hepatic tissue exposure.

### 3.7. ADME/T Profile and Drug-Likeness Assessment

The ADME/T analysis is a crucial pharmacokinetic context for interpreting the structure-based findings of the present study. Although the docking, MM-GBSA, and molecular dynamics analyses provide complementary computational information for prioritizing compounds with predicted structural compatibility, their translational relevance also depends on physicochemical properties, membrane permeability, solubility, absorption, distribution, metabolism, and safety-related parameters [51]. In this respect, the PPIs studied showed physicochemical and drug-likeness profiles that are compatible with small molecule drug space, but some compounds were more or less different.

Among the PPIs, omeprazole, pantoprazole and rabeprazole have relatively balanced physicochemical profiles. Our predicted QPlogPo/w values were 2.2, 2.8 and 2.5, respectively, while QPlogS was between −2.3 and −2.9 and remained within the reference ranges we used for this QikProp analysis. Rabeprazole, as the most preferred PPI for the NLRP3 7PZC structure, has a predicted molecular weight of 359 Da, PSA of 73 Å^2^ and QPlogPo/w of 2.5 and QPlogS of −2.3. Furthermore, we did not predict a Rule-of-Five violation for rabeprazole. These in silico results indicate that rabeprazole possesses several physicochemical properties that fall within commonly used drug-likeness reference ranges. However, such predicted properties do not establish that sufficient unbound concentrations are achieved in plasma or hepatic tissue to engage the proposed inflammatory targets. Therefore, the ADME/T predictions should be considered complementary pharmacokinetic context rather than evidence of translational feasibility [52].

Lansoprazole also showed some good predicted pharmacokinetic properties. Its QPPCaco and QPPMDCK values were 5870 and 6250 nm/s, respectively, indicating high predicted membrane permeability in the applied model. Furthermore, the predicted human oral absorption was 100%, and no Rule-of-Five violations were found. These are all very encouraging, as lansoprazole was the best PPI in both of the C5aR1 binding environments identified. Hence, docking and ADME/T results together indicate the potential for lansoprazole as a potential scaffold for further study. Still, some unusual values were obtained, such as a dipole moment of 26.2 D, globularity of 1.1 and ionization potential of 6.0 eV, which is outside of the QikProp reference range. These parameters should be taken cautiously and should be checked with the molecular structure, protonation state and input geometry of the model for ADME/T before making any mechanistic conclusions [53].

A different profile was found for avacopan. Even though lansoprazole produced the best MM-GBSA energies in the C5aR1 complexes with ΔGbind of −43.90 kcal/mol for 6C1R and −43.39 kcal/mol for 5O9H, its predicted ADME profile showed much higher lipophilicity and lower aqueous solubility than PPIs’. QPlogPo/w of 7.2 was much higher than the reference, while QPlogS and CIQPlogS of −9.3 and −9.5 predicted very low aqueous solubility. Avacopan also showed two Rule-of-Five violations. As such, its very good value for protein binding energy should be taken into account, as well as these predicted physicochemical issues.

The predicted toxicity parameters also have to be interpreted with caution. QPlogHERG values ranged from −0.4 for lansoprazole to −6.2 for avacopan, whereas rabeprazole was −5.9. Since these are not real cardiac electrophysiological data but computational predictions, they are screening indicators, not direct evidence of cardiotoxicity or cardiac safety. Similarly, the predicted number of metabolic transformations ranged from one for lansoprazole to seven for omeprazole, and so the predicted metabolic susceptibility is dependent upon the compound, which would need to be confirmed in experiments.

In general, the ADME/T results and the docking, MM-GBSA and molecular dynamics results together make the candidate-prioritization approach of this study more realistic. Rabeprazole is particularly interesting because it exhibits a good expected interaction with the NLRP3 7PZC structure and has a relatively balanced drug-likeness profile, whereas lansoprazole shows good C5aR1 docking and a high predicted membrane permeability and oral absorption. However, the ADME/T parameters presented here are only estimates and not experimental data for drug effect or toxicology. Thus, we advocate the study of rabeprazole and lansoprazole as a next step in the study of target engagement, cellular, pharmacokinetic and safety of AILDs.

Although several predicted ADME/T parameters fell within commonly used reference ranges, these predictions do not establish target-site exposure. In particular, predicted oral absorption or membrane permeability should not be interpreted as evidence that therapeutically administered PPIs reach sufficient unbound concentrations in plasma or liver to engage the investigated inflammatory proteins.

### 3.8. Limitations of the Study 

Although this study provides a structure-based rationale for the potential repurposing of PPIs, several limitations should be acknowledged. First, molecular docking calculations were primarily performed using static receptor conformations and therefore cannot fully reproduce receptor flexibility, induced-fit rearrangements, or conformational changes that may occur upon ligand binding. Although the use of multiple experimentally determined conformations for NLRP3 and C5aR1 partially accounts for receptor-state variability, these structures represent only a limited subset of the conformational ensemble accessible under physiological conditions. A 100 ns molecular dynamics simulation was therefore performed for the prioritized rabeprazole–NLRP3 (7PZC) complex to examine its time-dependent behavior and predicted complex stability. Nevertheless, this simulation was restricted to a single prioritized complex and cannot eliminate the inherent limitations associated with the static docking calculations performed for the remaining ligand–target combinations. Future studies employing replicate and longer-timescale MD simulations across additional PPI–target complexes would provide a more comprehensive assessment of receptor flexibility and binding-pose persistence. Second, although MM-GBSA is widely used as a post-docking rescoring approach to estimate ligand–protein binding free energy, it remains an approximate endpoint free-energy method. Therefore, the favorable ΔG_bind_ value observed for the rabeprazole–NLRP3 (7PZC) complex should be interpreted as supporting predicted complex stability and candidate prioritization rather than confirming biological inhibition. Because MM-GBSA calculations were limited to the top-ranked rabeprazole–NLRP3 (7PZC) complex, future studies should extend free-energy and molecular dynamics analyses to other promising PPI–target combinations. Third, solvent effects represent an additional limitation of the present computational framework. Crystallographic water molecules were removed during receptor preparation, and explicit solvent molecules were not retained during the docking calculations. Consequently, potentially important water-mediated hydrogen bonds, displacement of structured water molecules, and solvent-dependent stabilization or destabilization of particular ligand poses may not be fully represented in the docking scores. Although the subsequent MM-GBSA calculations incorporate solvation contributions through the generalized Born implicit-solvent model, this treatment cannot fully reproduce the molecular organization and dynamic behavior of explicit water molecules within individual binding pockets. Therefore, the predicted interaction patterns and energetic rankings should be interpreted as computational approximations rather than exact representations of ligand recognition under physiological aqueous conditions.

An additional limitation concerns the interpretation of favorable docking across multiple structurally unrelated targets. Because PPIs contain hydrophobic and aromatic structural features and several of the investigated binding sites possess substantial hydrophobic character, part of the observed cross-target docking compatibility may reflect nonspecific hydrophobic accommodation rather than selective molecular recognition. Therefore, favorable predictions for TLR4/MD-2, NLRP3, and C5aR1 cannot by themselves establish genuine polypharmacology. The present study also did not include systematic counter-screening against unrelated proteins or alternative hydrophobic pockets. Such counter-screening, together with experimental binding and functional assays, will be necessary to distinguish target-specific interactions from general ligand promiscuity in future studies.

The −6.0 kcal/mol docking-score criterion used in this study should also be considered an operational screening value rather than a universal activity threshold. Because docking scores are dependent on the target, receptor conformation, ligand characteristics, and scoring algorithm, this cutoff was used only to support within-protocol candidate prioritization and was not used to infer experimental binding affinity or biological activity [54,55]. Finally, because the present study was designed as a structure-based candidate-prioritization analysis, experimental validation using in vitro and in vivo models will be required to determine whether the predicted interactions translate into measurable modulation of TLR4/MD-2, NLRP3, or C5aR1 signaling.

The reference ligands included in this study should not be regarded as pharmacological benchmarks for the PPIs. TAK-242, MCC950, and avacopan differ substantially from PPIs in chemical structure, optimization history, experimentally established mechanism of action, and target-engagement characteristics. Moreover, docking scores are scoring-function-dependent estimates of predicted ligand–receptor poses and are not equivalent to experimentally measured binding affinities, IC_50_/EC_50_ values, or functional inhibitory potency. Consequently, numerical docking-score differences between PPIs and these reference compounds were used only to provide structural and protocol-specific context and were not used to establish pharmacological superiority, equivalence, or inferiority. Direct potency comparisons would require appropriately designed experimental binding and functional assays.

A major translational limitation of the present study is the absence of experimentally determined concentration–response relationships for PPI interactions with TLR4/MD-2, NLRP3, and C5aR1. Docking scores and MM-GBSA energies cannot be directly translated into pharmacologically required concentrations. Moreover, clinically administered PPIs are extensively metabolized and highly protein-bound, which substantially reduces the freely available systemic drug fraction. Quantitative unbound hepatic exposure data are also insufficient to determine whether the proposed targets would be exposed to pharmacologically relevant concentrations in vivo. Consequently, the present study cannot establish that the predicted interactions are achievable under standard therapeutic dosing conditions.

## 4. Materials and Methods

### 4.1. Study Design and Selection of Target Proteins 

**Figure 12:**
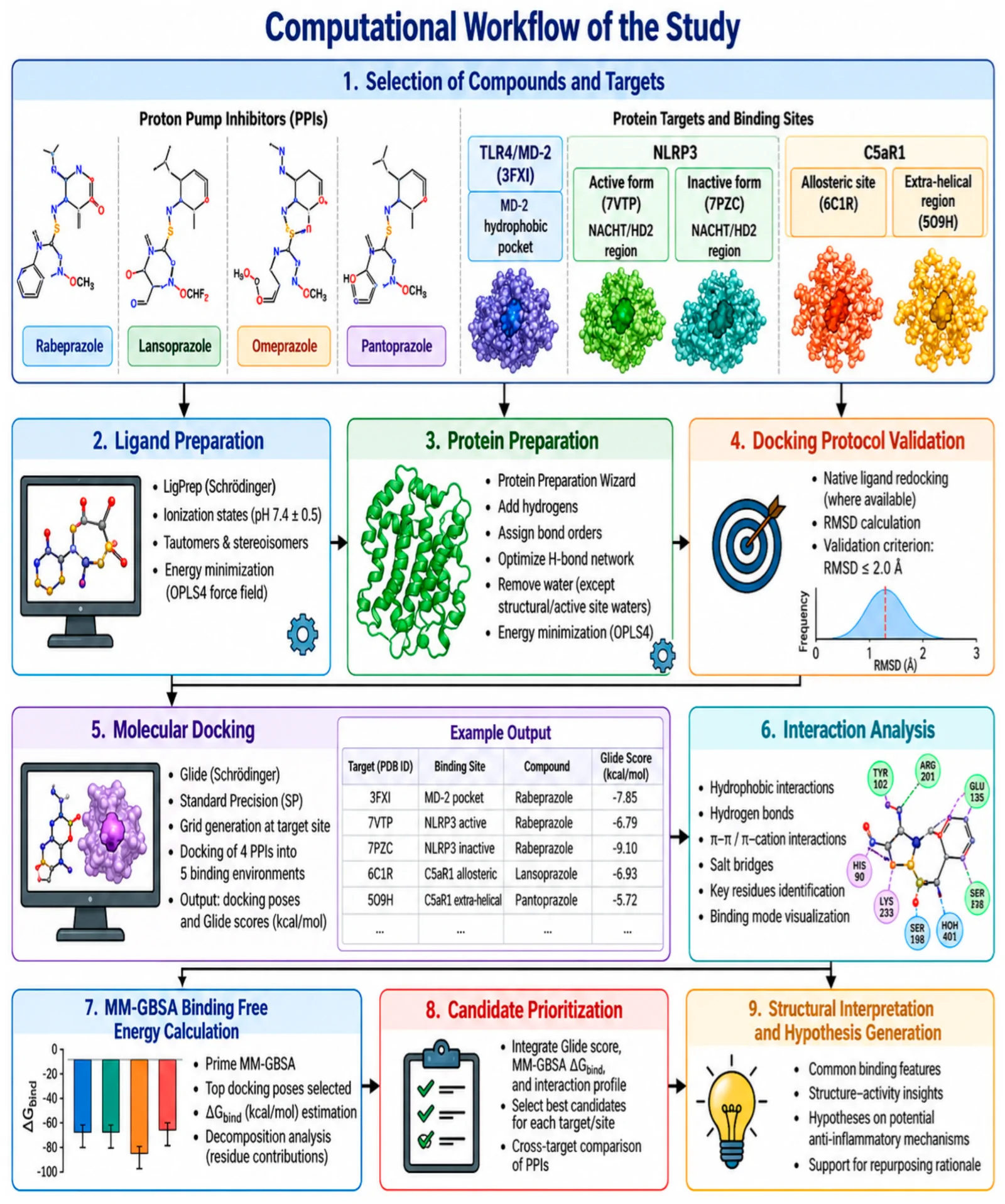
Study’s computational workflow.

The binding affinities and binding modes of four clinically approved Proton Pump Inhibitors (PPIs)—omeprazole, pantoprazole, lansoprazole, and rabeprazole—were analyzed using in silico molecular docking against key protein targets implicated in hepatic inflammation and immune-mediated liver injury, such as the TLR4/MD-2 complex (PDB ID: 3FXI), the NLRP3 inflammasome (PDB IDs: 7VTP and 7PZC), and the C5aR1 receptor (PDB IDs: 6C1R and 5O9H). The study’s computational workflow is given in Figure 12.

For the TLR4/MD-2 complex, the crystal structure 3FXI—representing a lipopolysaccharide (LPS)-bound active conformation—was selected to evaluate the competitive inhibitory potential of PPIs within the same hydrophobic pocket utilized by LPS [29]. TAK-242 was included as a pharmacologically established TLR4-pathway reference for contextual comparison. Because its established inhibitory mechanism differs from competitive occupation of the extracellular MD-2 pocket, it was not treated as a potency-matched positive control for MD-2 binding [28,30].

To encompass the distinct conformational states of the NLRP3 inflammasome, two Cryo-EM structures were utilized. The 7VTP structure, representing the hexameric/dodecameric oligomerized state, was chosen to assess interactions at the protomer–protomer interfaces critical for inflammasome activation [31]. Conversely, the 7PZC structure, co-crystallized with the candidate ligand CRID3, provided a precisely defined allosteric binding pocket [32]. MCC950, an experimentally established potent and selective NLRP3 inhibitor, was included as a structural and pharmacological reference. Its docking score was used only for protocol-specific contextual comparison and not as a surrogate for its experimentally measured potency [33,34,35]. 

For the C5aR1 receptor, two distinct topological models were included to thoroughly map potential antagonism. The 6C1R complex, co-crystallized with the allosteric candidate ligand avacopan (CCX168) and the orthosteric antagonist PMX53, facilitated the study of the validated allosteric pocket located at the upper region of the transmembrane bundle [36]. Concurrently, the 5O9H structure, complexed with the extra-helical antagonist NDT9513727, was employed to explore inhibition mechanisms external to the helical bundle (between TM3–TM4–TM5) [38]. Avacopan was utilized as the positive control in these analyses [37,39]. 

For descriptive screening and candidate prioritization, a docking score of ≤ −6.0 kcal/mol was used as an operational threshold. This value was selected because docking scores around or below −6.0 kcal/mol have been employed in previous and recent virtual-screening studies as an initial criterion for retaining compounds for subsequent computational evaluation [40]. However, this cutoff is not universally applicable and should not be interpreted as a validated boundary between active and inactive ligands since docking-score distributions and their relationship with experimental affinity depend on the scoring function, docking software, target protein, binding-site characteristics, and ligand set. Accordingly, the −6.0 kcal/mol value was used in the present study only as a pragmatic screening criterion to facilitate within-protocol prioritization, while candidate interpretation additionally considered binding-pose characteristics, ligand efficiency, interaction-energy components, MM-GBSA calculations, and molecular dynamics analysis [56,57,58].

### 4.2. Procedure for Docking Calculations

All molecular modeling, docking simulations, and visualization procedures were conducted using the Maestro Molecular Modeling Platform (11 september 2026) [59].

Protein Preparation: Target crystal structures were retrieved from the Protein Data Bank (PDB) and processed using the Protein Preparation Wizard [60]. Hydrogen bond networks were optimized, crystallographic water molecules were removed, and the structures were energy-minimized using the OPLS4 force field. Binding regions were defined based on the co-crystallized ligands and/or established active or allosteric sites of the target proteins [60,61].

Ligand Preparation: The three-dimensional structures of the PPI molecules and reference candidate ligands were generated and geometrically optimized using the LigPrep module [62] under physiological pH conditions to assign appropriate protonation and tautomeric states and to identify energetically favorable conformations. 

Grid Generation: Receptor grid boxes were centered on the established active and allosteric sites with the following coordinates: 3FXI (X: 28.01, Y: −2.83, Z: 6.00); 7VTP (X: 111.73, Y: 81.77, Z: 94.38); 7PZC (X: 189.77, Y: 192.94, Z: 114.06); 6C1R (X: 12.60, Y: 2.53, Z: −45.83); and 5O9H (X: 102.66, Y: −9.45, Z: 43.43). 

Docking and Validation: Molecular docking was performed using the Glide module in Standard Precision (SP) mode [63,64,65]. For receptor structures containing a co-crystallized ligand, the docking protocol was validated by native-ligand redocking for each receptor structure. The co-crystallized ligand was removed from its original binding site and redocked using the same receptor grid and docking parameters employed in the production calculations. The resulting redocked pose was superimposed onto the experimentally determined crystallographic pose, and the heavy-atom RMSD was calculated. The obtained RMSD values were 1.24 Å for 3FXI, 1.47 Å for 7VTP, 0.86 Å for 7PZC, 1.31 Å for 6C1R, and 1.58 Å for 5O9H. All values were below the predefined 2.0 Å criterion, indicating satisfactory reproduction of the crystallographic binding poses under the applied docking protocol. 

**Figure 13:**
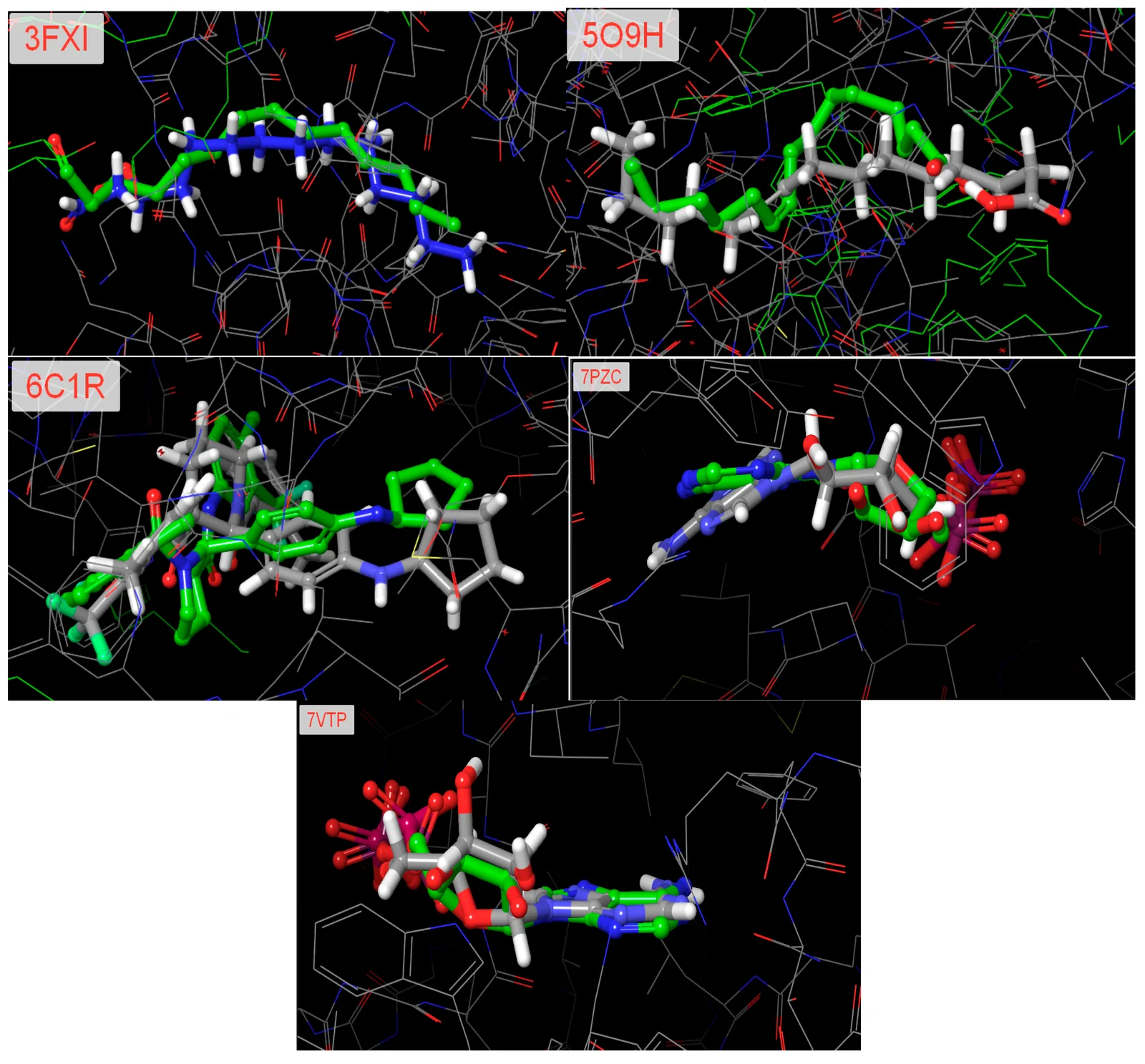
Presentation of the corresponding crystallographic/redocked pose overlays.

The individual RMSD values are summarized in Table 8, and the corresponding crystallographic/redocked pose overlays are presented in Figure 13.

Data Analysis and Scoring Parameters

Docking results were evaluated using docking-derived scoring parameters generated by Glide. Docking Score was used as the primary estimate of predicted docking score, with more negative values indicating more favorable predicted ligand–protein binding. Glide Ligand Efficiency was used to normalize the docking score according to ligand size. The Glide evdw and ecoul terms represented van der Waals and electrostatic interaction components, respectively. Glide einternal reflected the internal strain or conformational penalty of the ligand upon binding, with lower values suggesting more favorable ligand conformations. Molecular interaction profiles, including hydrogen bonds, π–π interactions, salt bridges, and hydrophobic contacts, were also recorded for the top-ranked poses. Additional Glide output parameters, including Glide hbond, emodel, energy, and pose number, were used when relevant to support pose selection and interaction interpretation.

ADME/T

In silico ADME/T profiling was performed using the QikProp module of Schrödinger to provide physicochemical and pharmacokinetic context for the computationally prioritized PPIs. The evaluated descriptors included predicted lipophilicity, aqueous solubility, membrane permeability, oral absorption, distribution-related parameters, and commonly used drug-likeness criteria. These values were treated as computational predictions and were not interpreted as experimentally established pharmacokinetic or safety properties. The Qik-prop module [66] of the Schrodinger program was used in order to make predictions about the effects and reactions that chemicals produce in human metabolism.

### 4.3. MM-GBSA Free Energy Calculations

To complement the docking analysis with an additional energetic assessment, MM-GBSA calculations were expanded to the top-ranked PPI–target complex identified for each investigated receptor structure. Accordingly, five complexes were evaluated: rabeprazole–TLR4/MD-2 (3FXI), rabeprazole–NLRP3 (7VTP), rabeprazole–NLRP3 (7PZC), lansoprazole–C5aR1 (6C1R), and lansoprazole–C5aR1 (5O9H). MM-GBSA calculations were performed using the Prime module of Schrödinger with the VSGB solvation model and OPLS4 force field. The calculated ΔGbind values and their individual energetic components were used for comparative assessment of the modeled complexes within the applied computational framework. These values were treated as computational estimates and not as experimentally determined binding free energies or measures of inhibitory potency. Molecular Mechanics/Generalized Born Surface Area (MM-GBSA) calculations were performed using the Prime module of Schrödinger. The binding free energy (ΔG_bind) and individual energy components were calculated using the VSGB solvation model and the OPLS4 force field.

### 4.4. Molecular Dynamics (MD) Simulation

We employed MD modeling to evaluate the effectiveness of the interaction between the protein and the ligand molecule, with the greatest performance achieved by examining their affinity for one another [67]. The MD simulation was carried out using Schrödinger’s Desmond software [68]. The OPLS force field was used to start the simulation by minimizing the protein–ligand bond’s energy [69]. To produce a box of water, the compounds were positioned 10 Å apart inside an orthorhombic box. The system was then neutralized by adding a certain amount of Na^+^/Cl^−^ ions [68]. The study used exact data for temperature, pressure, time, and distance to investigate how a chemical and a protein interact. 310.15 K (37 °C) was the starting temperature, 9 Å was the cut-off, 1.01325 bar was the pressure, and 2 fs was the time step [69]. While the Van der Waals and electrostatic interactions were examined, these values remained unchanged for 100 ns. A number of parameters were analyzed, including the bonds formed, the properties of the ligand, root mean square fluctuations (RMSFs), Radius of gyration (Rg), and root mean square deviation (RMSD).

## 5. Conclusions

In conclusion, this study suggests that clinically established proton pump inhibitors, particularly rabeprazole and lansoprazole, may exhibit a potential multi-target modulatory profile against TLR4/MD-2, NLRP3, and C5aR1 signaling pathways, which are relevant to hepatic inflammation. The structural features of the benzimidazole scaffold and molecule-specific side chains may contribute to immunomodulatory properties beyond acid suppression. These in silico findings provide a rationale for further experimental evaluation of PPIs as potential adjunctive candidates in autoimmune liver diseases, including AIH, PBC, and PSC. However, the present computational results do not establish that the predicted interactions occur at clinically achievable PPI exposures. Experimental determination of target-binding affinity and functional potency, followed by comparison with clinically achievable unbound plasma and hepatic tissue concentrations, will be essential to determine whether these predicted interactions are pharmacokinetically and biologically feasible before any therapeutic interpretation can be made.

## Figures and Tables

**Table 1:** Molecular docking parameters of proton pump inhibitors and the reference candidate ligand TAK-242 with the TLR4/MD-2 (3FXI) complex.

TLR4/MD-2 (3FXI)	Docking Score	Glide Ligand Efficiency	Glide Hbond	Glide Evdw	Glide Ecoul	Glide Emodel	Glide Energy	Glide Einternal	Glide Posenum
Lansoprazole	−7.41	−0.30	0.00	−36.29	−3.94	−65.43	−40.23	1.89	157
Omeprazole	−7.05	−0.29	0.00	−24.41	−26.98	−81.08	−51.40	1.66	147
Pantoprazole	−5.98	−0.23	−0.48	−22.07	−16.33	−59.98	−38.40	3.39	387
Rabeprazole	−7.85	−0.31	−0.61	−39.25	−8.94	−68.32	−48.19	5.44	103
TAK-242	−6.04	−0.26	0.00	−31.98	−2.46	−46.39	−34.43	2.21	31

**Table 2:** Molecular docking parameters of proton pump inhibitors and the reference candidate ligand MCC950 with the NLRP3 (7VTP) active conformation.

NLRP3 (7VTP)	Docking Score	Glide Ligand Efficiency	Glide Hbond	Glide Evdw	Glide Ecoul	Glide Emodel	Glide Energy	Glide Einternal	Glide Posenum
Lansoprazole	−6.76	−0.27	0.00	−38.55	−3.95	−54.73	−42.51	0.69	145
Omeprazole	−6.34	−0.26	−0.40	−37.45	−4.39	−51.24	−41.84	5.60	296
Pantoprazole	−5.59	−0.21	−0.41	−33.13	−8.63	−51.92	−41.75	5.09	107
Rabeprazole	−6.79	−0.27	−0.16	−45.13	−2.26	−60.17	−47.40	5.83	278
MCC950	−6.25	−0.22	−0.32	−40.14	−5.58	−64.09	−45.72	4.65	109

**Table 3:** Molecular docking parameters of proton pump inhibitors and the reference candidate ligand MCC950 with the NLRP3 (7PZC) inactive conformation.

NLRP3 (7PZC)	Docking Score	Glide Ligand Efficiency	Glide Hbond	Glide Evdw	Glide Ecoul	Glide Emodel	Glide Energy	Glide Einternal	Glide Posenum
Lansoprazole	−9.04	−0.36	−0.29	−38.72	−8.02	−66.65	−46.74	1.97	3
Omeprazole	−7.16	−0.30	0.00	−40.02	−2.89	−54.07	−42.91	6.66	16
Pantoprazole	−6.48	−0.25	0.00	−40.92	0.25	−57.59	−40.66	4.28	69
Rabeprazole	−9.10	−0.36	−0.28	−40.83	−7.49	−65.62	−48.32	4.33	338
MCC950	−6.40	−0.23	−0.10	−44.63	0.30	−65.42	−44.33	4.54	386

**Table 4:** Molecular docking parameters of proton pump inhibitors and the reference ligand avacopan at the C5aR1 (6C1R) allosteric pocket. Avacopan was included for structural and protocol-specific computational comparison; docking-score differences should not be interpreted as relative binding affinity or pharmacological potency.

C5aR1 (6C1R)	Docking Score	Glide Ligand Efficiency	Glide Hbond	Glide Evdw	Glide Ecoul	Glide Emodel	Glide Energy	Glide Einternal	Glide Posenum
Lansoprazole	−6.93	−0.28	−0.49	−29.05	−9.30	−53.08	−38.36	1.98	350
Omeprazole	−5.85	−0.24	0.00	−34.22	−4.36	−51.34	−38.58	1.08	327
Pantoprazole	−5.87	−0.23	−0.32	−30.77	−7.12	−52.07	−37.89	2.03	253
Rabeprazole	−6.46	−0.26	−0.45	−31.26	−8.66	−55.35	−39.92	0.84	164
Avacopan	−7.94	−0.19	−0.32	−46.00	−9.28	−75.77	−55.29	21.78	71

**Table 5:** Molecular docking parameters of proton pump inhibitors and the reference ligand avacopan at the C5aR1 (5O9H) extra-helical region. Avacopan was included for structural and protocol-specific computational comparison; docking-score differences should not be interpreted as relative binding affinity or pharmacological potency.

C5aR1 (5O9H)	Docking Score	Glide Ligand Efficiency	Glide Hbond	Glide Evdw	Glide Ecoul	Glide Emodel	Glide Energy	Glide Einternal	Glide Posenum
Lansoprazole	−6.54	−0.26	0.00	−30.95	−3.11	−46.58	−34.05	1.27	94
Omeprazole	−5.72	−0.24	−0.43	−33.37	−4.26	−48.27	−37.63	1.68	325
Pantoprazole	−5.21	−0.20	0.00	−34.07	−3.78	−47.77	−37.84	3.58	372
Rabeprazole	−5.94	−0.24	0.00	−39.32	−2.97	−54.65	−42.29	4.48	206
Avacopan	−8.67	−0.21	0.00	−47.48	−0.03	−65.18	−47.51	7.60	210

**Table 6:** Calculated MM-GBSA binding free energy components for all proteins with the best molecule. (kcal/mol).

Energy Component	7PZC with Rabeprazole	6C1R with Lansoprazole	5O9H with Lansoprazole	3FXI with Rabeprazole	7VTP with Rabeprazole
ΔG_bind_ (Total Binding)	−56.97	−43.90	−43.39	−53.96	−36.69
ΔG_bind_,_SolvGB_	−162.23	44.72	96.04	−40.82	208.11
ΔG_bind_,_vdW_	−46.64	3.42	0.36	4.74	7.10
ΔG_bind_,_Lipo_	−20.30	−0.92	−0.04	−0.45	−0.81
ΔG_bind_,_Coulomb_	168.65	−10.71	−12.25	−20.49	−16.80
ΔG_bind_,_Covalent_	5.39	−0.65	−1.41	−2.39	−1.80
ΔG_bind_,_Hbond_	−0.74	0.00	0.00	0.00	0.00
ΔG_bind_,_Packing_	−1.09	−45.26	−87.84	49.24	−181.05
ΔG_bind_,_SelfCont_	0.00	−34.50	−38.24	−43.78	−51.43

ΔG_bind_: Total binding free energy; Solv GB: Solvation/dissolving contribution calculated using the Generalized Born model; vdW: Van der Waals interaction energy; Lipo: Lipophilic (hydrophobic) interaction contribution; Coulomb: Electrostatic interaction energy; Covalent: Covalent contribution from bound interactions such as bond length/angle; Hbond: Hydrogen bond contribution; Packing: Geometric packing fit contribution; SelfCont: Internal contact energy of the ligand.

**Table 7:** ADME properties of molecule.

	Omeprazole	Pantoprazole	Rabeprazole	Avacopan	Lansoprazole	Reference Range
mol_MW	345	383	359	582	369	130–725
dipole (D)	3.2	5.4	3.3	2.4	26.2	1.0–12.5
SASA	600	621	650	870	303	300–1000
FOSA	321	210	279	443	230	0–750
FISA	80	81	80	64	24	7–330
PISA	190	225	281	238	0	0–450
WPSA	10	105	10	125	49	0–175
volume (A^3^)	1064	1084	1145	1675	551	500–2000
donorHB	1	1	1	2	0	0–6
accptHB	8	8	9	7	4	2.0–20.0
glob (Sphere = 1)	0.8	0.8	0.8	0.8	1.1	0.75–0.95
QPpolrz (A^3^)	35.0	35.5	37.2	59.9	15.9	13.0–70.0
QPlogPC16	10.3	10.2	11.7	15.6	5.1	4.0–18.0
QPlogPoct	17.1	17.9	18.3	26.9	23.6	8.0–35.0
QPlogPw	13.7	13.9	14.8	12.1	4.1	4.0–45.0
QPlogPo/w	2.2	2.8	2.5	7.2	1.2	−2.0–6.5
QPlogS	−2.3	−2.9	−2.3	−9.3	−0.3	−6.5–0.5
CIQPlogS	−4.0	−4.7	−3.9	−9.5	−3.9	−6.5–0.5
QPlogHERG	−5.0	−5.5	−5.9	−6.2	−0.4	*
QPPCaco (nm/sec)	69	68	69	2447	5870	**
QPlogBB	−0.5	−0.3	−0.7	−0.1	0.5	−3.0–1.2
QPPMDCK (nm/sec)	1017	3301	1008	6330	6250	**
QPlogKp	−1.8	−1.6	−1.2	−1.4	−2.0	Kp in cm/hr
IP (ev)	8.8	9.0	8.9	8.2	6.0	7.9–10.5
EA (eV)	0.7	0.9	0.7	0.5	0.0	−0.9–1.7
#metab	7	6	6	6	1	1–8
QPlogKhsa	−0.3	−0.3	−0.4	1.6	−0.7	−1.5–1.5
Human Oral Absorption	2	3	3	1	3	-
Percent Human Oral Absorpt.	73	76	75	100	100	***
PSA	73	79	73	65	21	7–200
RuleOfFive	0	0	0	2	0	Maximum is 4
RuleOfThree	1	0	0	1	0	Maximum is 3
Jm	27.1	12.0	115.1	0.0	1809.6	**-**

* corcern below −5, ** <25 is poor and >500 is great, *** <25% is poor and >80% is high.

**Table 8:** Validation of the molecular docking protocol by re-docking of co-crystallized ligands.

Target	PDB ID	RMSD (Å)	Acceptance Criterion
TLR4/MD-2	3FXI	1.24	<2.0 Å
NLRP3	7VTP	1.47	<2.0 Å
NLRP3	7PZC	0.86	<2.0 Å
C5aR1	6C1R	1.31	<2.0 Å
C5aR1	5O9H	1.58	<2.0 Å

## Data Availability

All data generated or analyzed during this study are included in this article. The protein structures used in the analyses are publicly available from the Protein Data Bank under the accession codes 3FXI, 7VTP, 7PZC, 6C1R, and 5O9H. Further inquiries can be directed to the corresponding author.
